# Defense of pyrethrum flowers: repelling herbivores and recruiting carnivores by producing aphid alarm pheromone

**DOI:** 10.1111/nph.15869

**Published:** 2019-05-31

**Authors:** Jinjin Li, Hao Hu, Jing Mao, Lu Yu, Geert Stoopen, Manqun Wang, Roland Mumm, Norbert C. A. de Ruijter, Marcel Dicke, Maarten A. Jongsma, Caiyun Wang

**Affiliations:** ^1^ Key Laboratory of Horticultural Plant Biology Ministry of Education Huazhong Agricultural University Wuhan 430070 China; ^2^ Business Unit Bioscience Wageningen University and Research Droevendaalsesteeg 1 6708 PB Wageningen the Netherlands; ^3^ Wuhan Forestry and Fruit Tree Research Institute Wuhan Academy of Agricultural Science and Technology Wuhan 430070 China; ^4^ Hubei Insect Resources Utilization and Sustainable Pest Management Key Laboratory College of Plant Science and Technology Huazhong Agricultural University Wuhan 430070 China; ^5^ Laboratory of Cell Biology Wageningen University and Research Droevendaalsesteeg 1 6708 PB Wageningen the Netherlands; ^6^ Laboratory of Entomology Wageningen University and Research Droevendaalsesteeg 1 6708 PB Wageningen the Netherlands

**Keywords:** (*E*)‐β‐farnesene, *(E)‐β‐farnesene synthase*, aphid honeydew, cortex‐specific expression, false alarm

## Abstract

- (*E*)‐β‐Farnesene (EβF) is the predominant constituent of the alarm pheromone of most aphid pest species. Moreover, natural enemies of aphids use EβF to locate their aphid prey. Some plant species emit EβF, potentially as a defense against aphids, but field demonstrations are lacking.
- Here, we present field and laboratory studies of flower defense showing that ladybird beetles are predominantly attracted to young stage‐2 pyrethrum flowers that emitted the highest and purest levels of EβF. By contrast, aphids were repelled by EβF emitted by S2 pyrethrum flowers. Although peach aphids can adapt to pyrethrum plants in the laboratory, aphids were not recorded in the field.
- Pyrethrum's *(E)‐β‐farnesene synthase* (*EbFS*) gene is strongly expressed in inner cortex tissue surrounding the vascular system of the aphid‐preferred flower receptacle and peduncle, leading to elongated cells filled with EβF. Aphids that probe these tissues during settlement encounter and ingest plant EβF, as evidenced by the release in honeydew. These EβF concentrations in honeydew induce aphid alarm responses, suggesting an extra layer of this defense.
- Collectively, our data elucidate a defensive mimicry in pyrethrum flowers: the developmentally regulated and tissue‐specific EβF accumulation and emission both prevents attack by aphids and recruits aphid predators as bodyguards.

## Introduction

Flowers are crucial for plant reproduction and, thus, for Darwinian fitness. Although plant defenses have been extensively studied, this mostly relates to the vegetative stage (Howe & Jander, 2008). However, flower defenses against herbivores are currently gaining interest (Chrétien *et al*., 2018). For defense against herbivores, plants exploit mutualistic relationships with predators and parasitoids by inducing volatile organic compounds (VOCs) upon herbivore attack (Kessler & Baldwin, 2001). These natural enemies use induced plant VOCs to locate herbivores either based on rewarding experiences or by innate mechanisms (Kessler & Baldwin, 2001; Dicke & Baldwin, 2010), and herbivores also often avoid these induced VOCs during host selection (Dicke & Baldwin, 2010; Bruce & Pickett, 2011). Among such VOCs, the plant‐derived aphid alarm pheromone (*E*)‐β‐farnesene (EβF) (Vosteen *et al*., 2016) has a particular position. Apart from being a common plant ‘cry for help’ compound, usually as part of herbivore‐induced VOC blends (Mumm *et al*., 2003; Röse & Tumlinson, 2005; Harmel *et al*., 2007; Zhuang *et al*., 2012; Köllner *et al*., 2013), EβF is also the main or only alarm pheromone of the majority of aphid pest species (Francis *et al*., 2005a,b), and is used by aphid enemies to locate presumed herbivorous prey (Abassi *et al*., 2000; Beale *et al*., 2006; Harmel *et al*., 2007). Compared to the low amount of EβF (nanogram range) occasionally emitted by stressed aphids under attack (Joachim *et al*., 2013) and the short lifetime of this volatile in air (Holopainen & Blande, 2013), it has been suggested that aphids and their enemies probably require greater amounts of plant‐derived EβF (microgram range) for repellence and prey–habitat localization at least over long distances (Vosteen *et al*., 2016). As such, they could both directly repel aphid herbivores, and attract their natural enemies (Abassi *et al*., 2000; Verheggen *et al*., 2013). Such a double‐edged defensive mimicry system was studied with transgenic *Arabidopsis thaliana* plants that constitutively expressed a peppermint *E*β*F synthase* gene. These EβF‐emitting plants elicited potent effects on the behavior of the aphid *Myzus persicae* (alarm and repellent responses) and its parasitoid *Diaeretiella rapae* (an arrestant response), suggesting effective direct and indirect defense by the constitutively released compound (Beale *et al*., 2006).

However, since the early laboratory studies on aphid repellence (Gibson & Pickett, 1983) and predator attraction by prey‐ or plant‐derived EβF (Abassi *et al*., 2000; Francis *et al*., 2005a,b; Harmel *et al*., 2007), no field or ecological evidence of effective, alarm‐pheromone‐based plant defense has been presented (Avé *et al*., 1987; Kunert *et al*., 2010; Bruce *et al*., 2015; Vosteen *et al*., 2016). Multiple explanations for this have been proposed. First, EβF emissions, as in maize, cotton, sorghum and pine, are part of herbivore‐induced plant volatile blends (Mumm *et al*., 2003; Röse & Tumlinson, 2005; Harmel *et al*., 2007; Zhuang *et al*., 2012; Köllner *et al*., 2013), providing information on the presence of herbivores, but not specifically of stressed aphids. The other VOCs in the blend, such as (−)‐germacrene D and (*E*)‐β‐caryophyllene, can mask the alarm pheromone both to aphids and to their enemies (Dawson *et al*., 1984; Mostafavi *et al*., 1996; Abassi *et al*., 2000; Bruce *et al*., 2005). Second, aphids, and probably also parasitoids and predators, can discriminate between aphid‐ and plant‐derived EβF by monitoring changes in compound ratios: aphids are mainly responsive to bursts of EβF in an otherwise stable VOC background (mimicking the natural EβF emission of an attacked aphid by predators) and habituate to constant EβF exposure as revealed in studies with transgenic *A. thaliana* plants (Hatano *et al*., 2008; Kunert *et al*., 2010; de Vos *et al*., 2010; Joachim *et al*., 2013). Especially sudden or intermittent encounters with plant‐emitted EβF may therefore be required to effectively mimic an alarm signal of aphids.

The present study was triggered by the remarkably frequent visitation of pyrethrum flowers (*Tanacetum cinerariifolium*) by ladybird beetles as observed in pyrethrum fields in China despite the apparent absence of prey. We recorded that young flowers emit EβF as a dominant component, and hypothesized that this might explain the high densities of aphid predators and the absence of aphid prey on pyrethrum plants in the field. Here, we report field and laboratory studies that address the following research questions: Which plant developmental stages produce and emit EβF? Which flower tissues express the *E*β*F synthase* gene and which cells produce and store EβF? What is the effect of EβF storage and release on the behavior of aphids and their ladybird predators? Does EβF ingestion by aphids influence their behavior? By addressing these questions, our study reveals a specific and effective mode of flower defense in pyrethrum plants.

## Materials and Methods

### Plant and insect materials

Pyrethrum (*Tanacetum cinerariifolium*) of genotype ‘39’ with a high regeneration rate (Mao *et al*., 2013) was used and pyrethrum seeds for other pyrethrum plants of different genotypes were supplied by Nanbao Biological (Yunnan, China). Pyrethrum plants in pots were grown in glasshouse conditions at 20 ± 5°C with 12 h of light in Wuhan, China. Flowers at developmental stages S1–S6 (Wandahwa *et al*., 1996) (Fig. 2a) from three field‐grown genotypes were harvested, dissected, immediately flash frozen in liquid nitrogen and stored at −80°C for further analysis. For honeydew analysis, clonal pyrethrum plants from several genotypes generated by division propagation were grown in pots, in glasshouse conditions at 25 ± 2°C with 16 h of light in Wageningen, the Netherlands.

Ladybird beetle (*Coccinella septempunctata*) adults were obtained from fields in Yunnan province, kept in ventilated plastic boxes at 20°C, and fed with *Acyrthosiphon pisum* aphids. Green peach aphids (*M. persicae*) collected from lab‐grown tobacco seedlings were reared on 1‐month‐old *Nicotiana benthamiana* plants in a climate room (16 h light photoperiod; 60 ± 10% relative humidity; 25 ± 2°C) for honeydew collection. For the aphid dispersal assay, single *M. persicae* adults previously reared on *N. benthamiana* were inoculated on pyrethrum flowers and Chinese cabbage leaves for habituation and reproduction over at least 2 wk (de Vos *et al*., 2010).

### Survey of entomofauna

Several pyrethrum seed production fields in China were monitored for the presence and behavior of different insect species. The scale of the field and insect survey can be found in Supporting Information Methods S1. For ladybirds, the locations of larvae, pupae and adults on the plant were recorded. Numbers of small insects such as thrips hiding in the flowers were monitored as described (Yang *et al*., 2012). Representative specimens of different insect species were photographed, collected and stored in 70% alcohol, and identified to the species level in the laboratory.

### Headspace collection and volatile analysis of field samples by thermo‐desorption GC‐MS

For the field study, samples were placed in 1.0‐litre glass cuvettes closed with a Viton‐lined glass lid having an inlet and outlet. Flower peduncles were cut to the same length immediately preceding the measurements. Detailed information regarding the headspace collection system and samples can be found in Methods S2. Headspace was collected for 30 min after which samples and the two inlets and two outlets were all replaced for the next measurement. Any remaining VOCs were blown out of the bottle. The volatiles trapped in the outlet cartridges were analyzed as described by Yang *et al*. (2011). Raw data were processed using an untargeted metabolomics workflow (Mumm *et al*., 2016). Volatile compounds were putatively identified by comparing the obtained mass spectra with those in commercial and in‐house mass spectral libraries (NIST14) and by comparing the retention indices with those published in the literature. Retention indices were calculated based on a series of alkanes using a third‐order polynomial function.

### Headspace collection and analysis of intact pyrethrum plants and a single leaf by GC‐MS

For headspace analysis of insect‐free intact and mechanically damaged pyrethrum plants, plants were divided into four developmental stages: preflowering, S0–2, S2–4 and S4–6, with the numbers indicating the most dominant flowering stages. Six replications of each plant stage were used. For the mechanical‐damage treatment, flowering plants of the S2–4 stage were rapidly punctured 100 times with entomological pins on both the flower peduncle and receptacles and then immediately placed in the volatile collection vessels. Plant VOCs were then collected following a method similar to that described by Sun *et al*. (2013). Detailed information is given in Methods S3. A C8–C20 alkane series (Sigma‐Aldrich) and a dilution series of an EβF standard (1.6–200 ng μl^−1^) (Echelon Bioscience Inc., Salt Lake City, UT, USA) were analyzed regularly to provide references for calculation of the retention index, for quantitation of EβF and to monitor system performance. Compounds were identified by comparing the obtained mass spectra with those in commercial and in‐house mass spectral libraries (NIST14) and by comparing the retention indices with those published in the literature.

To investigate the dynamics of EβF, (*Z*)‐3‐hexen‐1‐ol (HO) and (*Z*)‐3‐hexenyl acetate (HA) release from single leaves after mechanical damage, we collected volatiles on solid phase micro‐extraction (SPME) fibers over different intervals after damage and analyzed them by GC‐MS (Zeng *et al*., 2016). Detailed information is given in Methods S3.

### Olfactory responses of ladybird beetles to different odors

To determine the response of ladybird beetle adults to odors of different developmental stages of clean or wounded pyrethrum plants, we used a 1.5 cm internal diameter Y‐tube olfactometer. Detailed information about the Y‐tube olfactometer system and experimental protocols can be found in Methods S4. If the individual entered 2 cm into one of the arms and remained in this arm for at least 30 s, it was considered to have made a choice. Serial dilutions of EβF, HO, HA and 6‐methyl‐5‐hepten‐2‐one (MHO) were also used to perform these experiments. Detailed commercial information about the products and concentrations used in the experiment can be found in Methods S4. In total, 100 µl diluted compound in a 2 ml glass tube was placed in a 1‐liter glass vessel and two air streams (3 l min^−1^) were led through the vessels containing the odor sources. Each insect was used only once. Replicate experiments were conducted on different days with different individual beetles. A χ^2^ test was used to determine significant differences between numbers of ladybird beetles choosing different odors.

### Isolation, characterization and functional expression of *E*β*F synthase* genes and promoter sequence from *T. cinerariifolium*

Total RNA and genomic DNA from pyrethrum S1 flower buds were isolated using Trizol and reverse transcribed as previously described (Zeng *et al*., 2016). Detailed information of gene and promoter sequence amplification and bioinformatics analysis is given in Methods S5. The complete open reading frame of the full‐length cDNA of the *E‐*β*‐farnesene synthase* (*EbFS*) gene was subcloned in‐frame and upstream of the (His)6‐tag of the pET6xHN‐C expression vector (www.clontech.com). Detailed information about expression and purification of the protein can be found in Methods S5. The protein concentration was determined according to the method of Bradford (1976). EbFS product assays were performed by incubating 28 μg purified enzyme with 10 μM (*E,E*)‐farnesyl diphosphate (FPP) or geranyl pyrophosphate (GPP) (Echelon Bioscience Inc.) in a total volume of 100 μl assay buffer, and then the sample was incubated and extracted as described by Yang *et al*. (2014). One microliter of the hexane extract was used for GC‐MS analysis. The GC‐MS programme was the same as that for headspace analysis of intact pyrethrum plants.

### Subcellular localization of *Tc*EbFS1 and *Tc*EbFS2

The open reading frames of *EbFS* without stop codon were fused downstream of the CaMV 35S promoter, and in frame with green fluorescent protein (GFP) in the pCAMBIA1302 vector using *Nco*I and *Stu*I cloning sites (Fermentas, Thermo Fisher Scientific Inc., Burlington, ON, Canada). One microgram of plasmid DNA per construct was used for PEG‐mediated *Arabidopsis* protoplast transformation as previously described (Yoo *et al*., 2007). Transient expression of GFP fusion proteins was observed after 16 h using a confocal laser scanning microscope (Zeiss) with fluorescence band filters of 620–750 nm for Chl imaging and 500–530 nm for GFP.

### Gene expression analysis using RT‐PCR

Plant RNA was isolated and reverse transcribed as described by Zeng *et al*. (2016), individually from flower heads of six developmental stages (S1–S6), and different parts of S3 flowers (flower peduncle, receptacle, ray flower, disk flower), young leaves and old leaves. Relative quantitation by real‐time gene expression analysis of *EbFS* and the reference gene *GAPDH* (Ramirez *et al*., 2012) was performed on an Applied Biosystems 7500 platform using SYBR green I with 6‐carboxyl‐X‐rhodamine (ROX) (Takara Biotechnology Co., Ltd, Dalian, China) as an internal standard according to the protocol of Zeng *et al*. (2016).

### Extraction and analysis of plant secondary metabolites

Terpene content of flower parts was extracted in hexane and analyzed by GC‐MS. After careful removal of the aphids, each pyrethrum S1 flower was cut into two parts (flower bud, peduncle), weighed and flash frozen in liquid nitrogen. Detailed information on sample extraction is given in Methods S6. One microliter of hexane was injected for GC‐MS analysis (equipment and settings were the same as those used for the honeydew analysis), and a full scan from 33 to 500 amu was performed. Three technical replicates of each of three biological replicates were performed. Identification and quantification of EβF were performed as that for headspace analysis of intact pyrethrum plants

Secondary metabolites stored in the different organs at various plant developmental stages were extracted and analyzed (detailed information is given in Methods S6).

### *EbFS* gene promoter fusions with *GUS* analyzed in chrysanthemum

The nucleotide sequence of a nearly 2.2 kb promoter region of the *EbFS* gene (deposited in GenBank: MF678596) was cloned by fusion primer and nested integrated PCR (FPNI‐PCR) (Wang *et al*., 2011) and subcloned into a modified PBI121 vector fused to the *GUS* reporter gene using *Bam*HI and *Sac*I restriction enzymes. *Chrysanthemum morifolium* ‘1581’ were transformed and transgenic plants were checked by PCR using a forward primer on the *EbFS* promoter sequence (PE‐F) and a reverse primer on the *GUS* gene (GUS‐R, sequences presented in Table S1). Expanded leaves with petiole and shoots were sampled from chrysanthemum plants to perform glucuronidase (GUS) analysis. GUS histochemical staining was carried out according to the manual of the *GUS* reporter gene staining kit (Sigma‐Aldrich). GUS‐stained shoots were embedded into resin according to the procedure described by Xiao *et al*. (2014) and cut into 8–10 μm slices with an Ultra‐Thin Semiautomatic Microtome (Leica RM2245, Wetzlar, Germany) and studied under a microscope (Nikon Eclipse 80i FL/DIC upright microscope) and photographs were taken using a high‐definition digital color camera (Nikon DS‐Fi1).

### Histochemistry and fluorescence microscopy

Fresh hand‐sections were made for the upper flower peduncle (S1) of pyrethrum and then immediately subjected to NADI (naphthol + dimethyl paraphenylenediamine) reagent. Sections were stained according to the procedure described by Caissard *et al*. (2004), then observed directly under the microscope (Olympus BX53, Tokyo, Japan). For sieve element observation, NADI‐stained sections were exposed to 0.1% aniline blue (Water Blue, Shanghai, China) in potassium phosphate buffer (0.1 M, pH 7.4) for 30 min and then rinsed for 10 min in this buffer. Aniline blue fluorescence was detected with an excitation light of 365 nm using a fluorescence microscope (Olympus BX53).

### Aphid behavior assay in response to early‐stage pyrethrum flowers

Aphids at fourth instar or young adult stages reared on cabbage plants or pyrethrum flowers were individually used to observe their first‐response behavior by continuously recording their position on a pyrethrum flower every 5 s. More details are given in Methods S7. Responses of aphids reared on cabbage plants or pyrethrum flowers following exposure to volatiles from five S2 flowers and synthetic EβF (1 μg μl^−1^ in hexane) were recorded. Air containing the tested odor was blown towards a single *M. persicae* aphid feeding on a cabbage leaf disk from a distance of 1 cm for 10 s. Detailed information is given in Methods S7. Aphids showing movement and leaving the feeding sites within 2 min were scored as a responder. Arcsin‐transformed data of responsive aphids were subjected to ANOVA and followed by the Duncan's multiple range test to assess differences in response behavior between the treatments.

### Aphid honeydew collection and volatile analysis

Approximately 50 aphids were inoculated on the flower bud (S1) of a fresh pyrethrum plant 12 h before honeydew collection. Fresh honeydew droplets were collected using a microcapillary with rubber balloon for suction and pressure and deposited into 50 μl hexane (containing 1.67 ng μl^−1^ carvone). Detailed information about honeydew collection and the GC‐MS system and programs can be found in Methods S8. Specific masses of EβF (41, 69, 93, 133, 204 *m*/*z*) were picked up for scanning mode running. Identification and quantification of EβF were performed same as that for headspace analysis of intact pyrethrum plants with a dilution series (0.0125–0.2 ng μl^−1^) of the authentic EβF standard.

### Aphid behavior assay in response to artificial honeydew

Ten micrograms of EβF standard was added to 1 ml 25% sucrose solution and vortexed for 30 min. On each occasion one individual *M. persicae* aphid (third or fourth nymphal stage) was carefully transferred to a fresh *N. benthamiana* leaf without prior aphid exposure. Each test was initiated when the aphid had resettled on the new leaf and folded its antennae backward. Either 200 nl honeydew (containing 0 or 10 ng μl^−1^ EβF) or synthetic EβF standard in hexane were applied directly onto the dorsum of each individual aphid. To mimic the brief resident time of honeydew before discharge, 200 nl artificial honeydew was also brought into very close range (<1 mm) for 5 or 3 s. The number of aphids moving within 2 min was recorded as a responder. The experiment contained three replications and 20 individual aphids were tested per replication. Arcsin‐transformed data of responsive aphids were subjected to ANOVA and followed by the Duncan's multiple range test to assess differences in response behavior between the treatments.

## Results

### Ladybird beetles predominate on young flowers in pyrethrum fields

**Figure 1:**
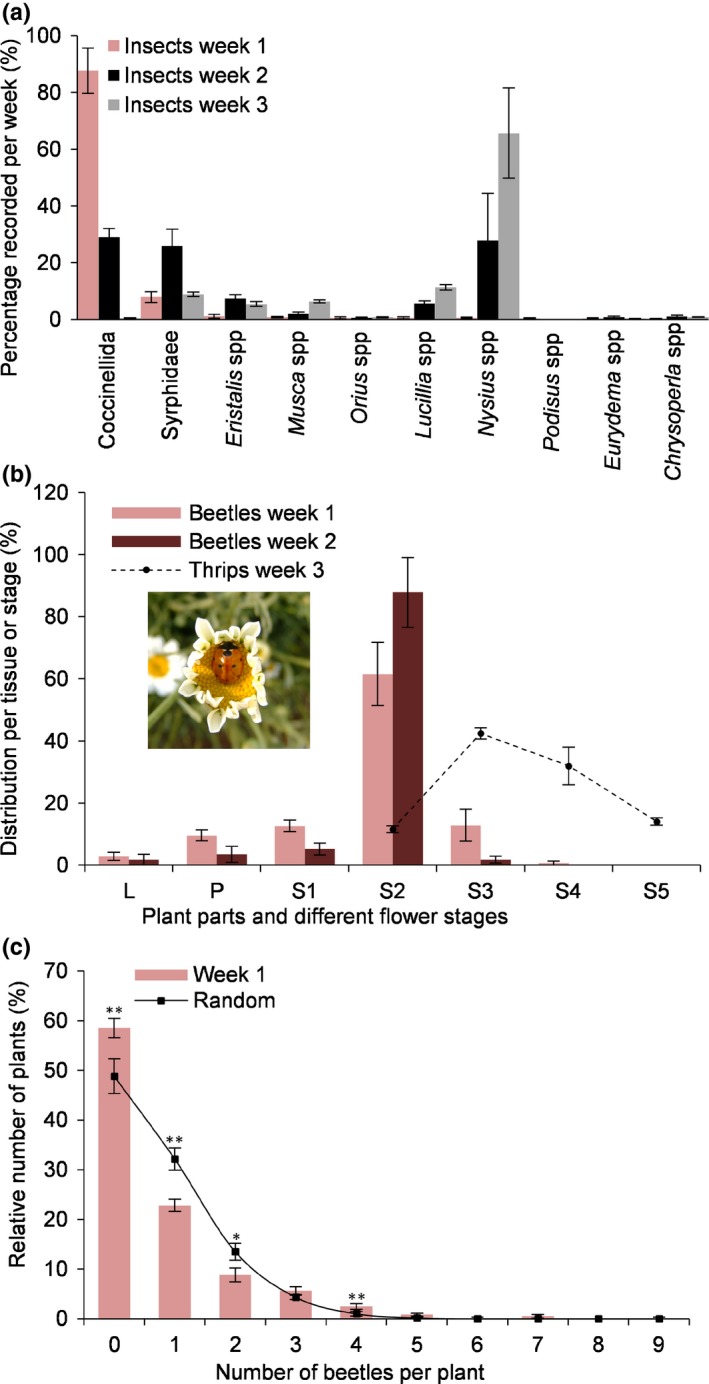
Insect distribution on field pyrethrum plants. (a) Relative abundance of different insect species visually recorded on pyrethrum plants during weeks 1–3 (week 1 represents early flowering stages S1–3; week 2 represents stages S2–4, and week 3 stages S4–5). Thrips were scored separately (Fig. 1b). Data are represented as mean ± SEM. The total number of individuals per week is 532, 393 and 2967 for weeks 1, 2 and 3, respectively. (b) Distribution of coccinellid beetles (mainly *Coccinella septempunctata*) and thrips (mainly *Frankliniella occidentalis*) across the different tissues and flower stages. Scored plant parts for beetles were leaves (L), peduncles (P) and flower stages 1–5 (S1–S5) in two fields scored in weeks 1 and 2. Beetle densities were 76 and 19 per 100 plants in weeks 1 and 2, respectively. Beetle density in week 3 was too low and is not shown here (two per 100 plants). Thrips were scored in week 3 with a highest density of eight thrips per flower on S3 flowers (Yang *et al*., 2012). Inset: ladybird beetles on an S2 flower head. Data are represented as mean ± SEM. (c) Distribution of the number of coccinellid beetles found per plant. The distribution recorded in week 1 is statistically significant compared to the calculated random distribution of the same number of insects (*t*‐test: *, *P* < 0.05; **, *P* < 0.01). All data in (a)–(c) are based on the assessment of six pools of 100 plants except for thrips (three pools). Data are represented as mean ± SEM.

**Figure 2:**
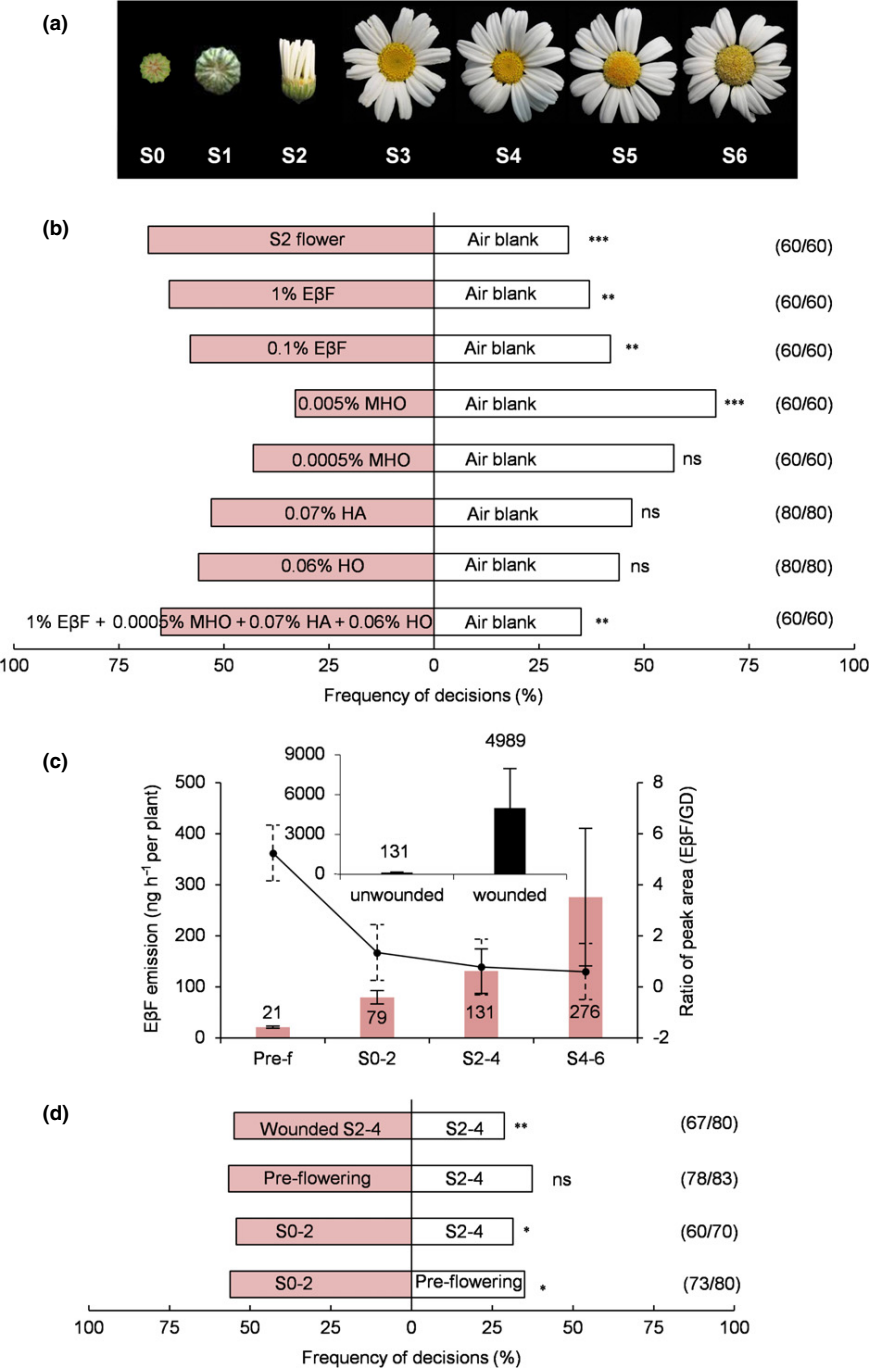
EβF emission and attraction of ladybird beetles. (a) Images of different pyrethrum flowering stages: Stage 0 (S0, younger bud), Stage 1 (S1, bud), Stage 2 (S2, ray flowers half open), Stage 3 (S3, first row of disk flowers open), Stage 4 (S4, half rows open), Stage 5 (S5, all rows open), Stage 6 (S6, overblown). (b) Response of ladybird beetles (*Coccinella septempunctata*) to volatile compounds against an air blank. Y‐tube olfactometer choice assays were performed with S2 flowers, different dilutions of pure compounds or a blend of (*Z*)‐3‐hexen‐1‐ol (HO), (*Z*)‐3‐hexenyl acetate (HA), 6‐methyl‐5‐hepten‐2‐one (MHO) and (*E*)‐β‐farnesene (EβF) (χ^2^ test; **, *P* < 0.01; ***, *P* < 0.001; ns, not significant). (c) Quantitated EβF emission during plant development (pink bars) and in response to artificial damage (S2–4 stage, inset). On the right axis, peak area ratios of EβF and germacrene D (GD). Data are represented as means ± SE (*n* = 4–5). (d) Y‐tube olfactometer choices of ladybird beetles in response to pyrethrum volatiles at different developmental stages or upon wounding. At the right, the number of responsive/total beetles (χ^2^ test, *, *P* < 0.05; **, *P* < 0.01; ns, not significant).

In a field survey of 600 plants in two fields in Yunnan province (China), 88% of the insects recorded on pyrethrum plants in the first week were ladybird beetles and *c. *8% syrphid flies (total number of insects was 532 in week 1 with young flower stages S1–S3, Figs 1a, 2a). The beetles were predominantly *Coccinella septempunctata*, and at a low frequency also *Hippodamia convergens* and *Harmonia axyridis*. In week 1 on average 0.77 ladybird beetles were found per plant, which at the local planting density corresponded to *c. *100 000 beetles ha^−1^. Later, ladybird beetle numbers dropped to much lower levels of 0.19 (week 2) and 0.02 (week 3) per plant, respectively. Final numbers were similar to other predators, namely *Orius* adults (Heteroptera) and *Chrysoperla* larvae (Neuroptera) (both 0.04 individuals per plant in week 2 and week 3) (Fig. 1a). Ladybird beetles were noted particularly on S2 flowers (half open ray flowers, cup‐shaped, Fig. 2a): 62% of all beetles on S2 flowers in week 1, and up to 88% in week 2 (Fig. 1b). Potential prey (Raza *et al*., 2015) such as the adults of *Nysius* sp. (Fig. 1a) and thrips (mainly *Frankliniella occidentalis*, Fig. 1b) preferred the pollen/seeds of the post‐anthesis later‐stage flowers and became dominant in later weeks by which time the beetles had left. In the majority of our field observations and sampling experiments across multiple years we did not observe any aphids.

### Ladybird beetles prefer pyrethrum flowers that emit more EβF in the field

Pyrethrum is an outcrossing, self‐incompatible plant species, and as a result in the field there was large phenotypic variation, each plant representing a different geno‐ and chemotype (Li *et al*., 2014). We observed that the distribution of beetles in the fields did not follow a random distribution, with some plants attracting more beetles (Fig. 1c). This offered the possibility to correlate the skewed distribution to any relevant constitutive and/or induced volatile factors emitted by the beetle‐preferred flowers. Pools of detached flowers at the S2 stage from 60 different plants scored for the presence of beetles were compared to pools of flowers on which beetles were not recorded. GC‐MS of flower headspace showed profiles dominated by EβF, HO and HA (Fig. S1). Two alarm pheromones, EβF and MHO, known to be released by aphids (Francis *et al*., 2005a,b) and ants (Stoeffler *et al*., 2007) respectively, one green leaf volatile HA and an unidentified compound were significantly more abundant in beetle‐preferred flowers. Four green leaf volatiles (including HO) were significantly more abundant in flowers without beetles (Table 1; Fig. S1). To test which of the compounds could explain the abundance of the beetles, a series of choice assays with ladybird beetles were done with pure compounds at concentrations that approximated the emission by flowers, taking into account the relative volatility of the diluted compounds in paraffin oil. EβF attracted the beetles (Fig. 2b), whereas MHO, HA and HO either did not have significant effects on attraction or became repellent at higher concentrations (MHO). When blending all four compounds, 65% of the beetles were attracted, which was similar to 63% attraction to EβF alone, and 68% to the full blend emitted by S2 flowers (Fig. 2b).

**Table 1:** Volatile compounds emitted from detached pyrethrum S2 flowers with/without ladybird beetles in the field.

Average abundance (×10^6^, *n* = 6, ±SE)		Quantitated mass	Paired compound ratios +/− beetles (%, *n* = 6)^a^	Paired *t*‐test	Kovat's index	Putative name
+ beetles	− beetles					
72.3 ± 18.3	47.9 ± 11.3	82	141 ± 20	0.05	1466	(*E*)‐β‐Farnesene
43.3 ± 3.7	38.0 ± 2.6	81	113 ± 4	0.02	1006	(*Z*)‐3‐Hexen‐1‐ol acetate
1.4 ± 0.2	0.8 ± 0.1	108	173 ± 24	0.02	988	6‐Methyl‐5‐hepten‐2‐one
1.0 ± 0.2	0.8 ± 0.2	57	124 ± 5	0.01	1501	Unknown
97.3 ± 6.0	106.3 ± 8.1	69	92 ± 2	0.02	858	(*E*)‐3‐Hexen‐1‐ol
10.6 ± 1.1	12.0 ± 1.0	57	88 ± 3	0.02	684	1‐Penten‐3‐ol
7.3 ± 1.4	11.5 ± 1.6	69	65 ± 8	0.02	799	(*Z*)‐3‐Hexenal
3.3 ± 0.4	4.7 ± 0.4	57	72 ± 8	0.03	866	2‐Hexen‐1‐ol

a +/−, with/without beetles.

### Ladybird beetles prefer early blooming stages that predominantly emit EβF and camphene

The headspace emissions of detached pyrethrum flowers contained green leaf volatiles that in major part probably resulted from the wounds inflicted by cutting peduncles. To test whether headspace emissions of intact plants contain EβF and attract ladybird beetles, we performed dynamic headspace analysis, and in parallel, Y‐tube olfactometer experiments using insect‐free preflowering plants and plants being predominantly in the S0–2, S2–4 and S4–6 flowering stages (Fig. 2a).

The headspace of the flowering plants mainly contained camphene, HA, isolongifolene, β‐longipinene, EβF and germacrene D in different ratios for different flowering stages (Fig. S2a). MHO and green leaf volatiles such as HO were not detected. Interestingly, on intact plants EβF and camphene were the highest peaks in the volatile blend only during the short period when most of the flower buds formed and began to open (S0–2). After that, germacrene D became the highest peak (Figs S2a,b). EβF emission increased from 21 ± 2 to 276 ± 135 ng h^−1^ per plant following the formation of the flower bud and blooming of the flower (Fig. 2c). Mechanical wounding increased this dramatically to over 5000 ng h^−1^ per plant mainly due to an apparent immediate release from internal stores (Figs 2c, S3a,b). To check which emissions could be induced by herbivores such as thrips (*Thrips tabaci*) or aphids (*M. persicae*), five pooled pyrethrum S2 flowers were infested followed by headspace analysis. Within the first 2 h of infestation, S2 flowers with 20 thrips per flower significantly increased HO emissions by a factor 30 compared to uninfested control flowers, but not HA or EβF (Fig. S3c). Infestation with 10 aphids per peduncle also increased HO (11‐fold), but also had no significant effect on HA and EβF emissions (Fig. S3c). In the field we found around two thrips per S2 flower in week 3 (Fig. 1b) (Yang *et al*., 2012), but on this basis it seems unlikely that thrips damage has played an important role in increasing EβF emission and promoting beetle attraction in field.

In Y‐tube olfactometer experiments with ladybird beetle adults, the S0–2 plants were significantly more attractive to beetles than preflowering plants and older flowering S2–4 plants (Fig. 2d). Germacrene D is probably formed by trichomes on maturing seeds inside the flowers that are also involved in the production of pyrethrins and sesquiterpene lactones (Ramirez *et al*., 2012). It is known to inhibit the alarm response of green peach aphids to EβF (Bruce *et al*., 2005), and it possibly modulated beetle preference for S0–2 plants over S2–4 plants (Fig. 2d), which emitted similar EβF levels, but with a higher germacrene D emission (Fig. 2c). Beetles also significantly preferred wounded S2–4 plants over unwounded ones (Fig. 2d).

Overall, these results show that pyrethrum emits EβF within a changing bouquet of volatiles during different flowering stages. As in the field, in a laboratory setting, ladybird beetles preferred the early‐flowering stage with the highest and purest EβF emission, and EβF was the only volatile attracting the beetles.

### Expression of *E‐*β*‐farnesene synthase* gene and accumulation of EβF occurs mainly in young flower peduncles

**Figure 3:**
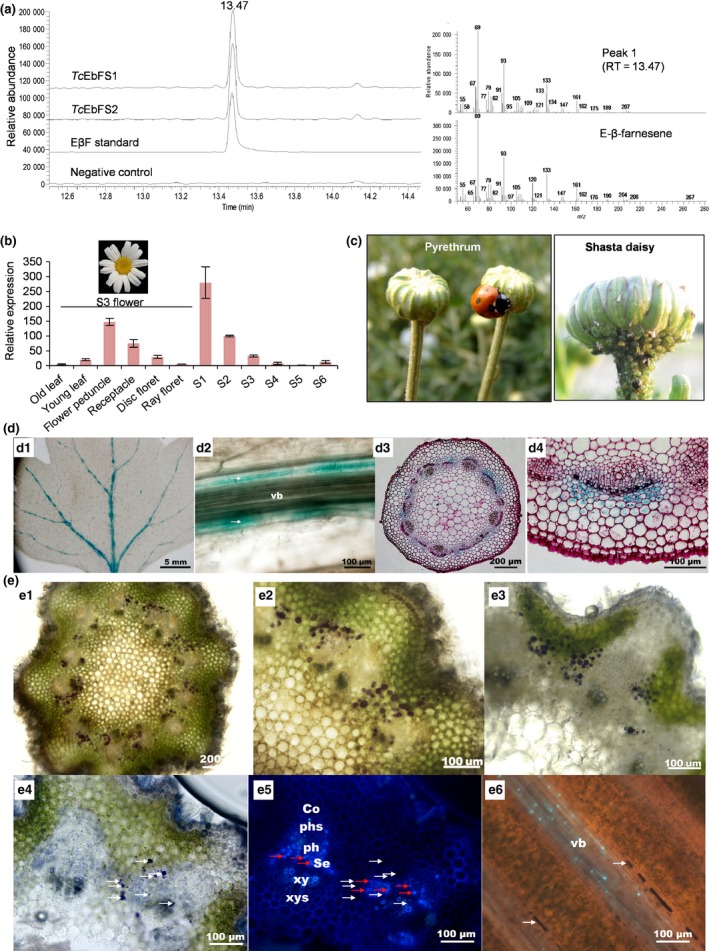
Localization analysis of *TcEbFS* gene expression and (E)‐β‐farnesene (EβF) in pyrethrum. (a) GC‐MS single ion traces (*m*/*z* 93) (left) of products formed by incubation of recombinant *Tc*EbFS1 or *Tc*EbFS2 protein with farnesyl diphosphate (FPP). Mass spectrum and retention index are compared to an authentic standard of (*E*)‐β‐farnesene. (b) Gene expression of *TcEbFS* in different organs and flower developmental stages. Floral parts of the S3 flower stage. Results of qRT‐PCR were normalized for *TcGAPDH*. Data are represented as means ± SE (*n* = 3). (c) Left: ladybird beetle searching on a flower bud of pyrethrum. Right: aphid infestation of a bud of shasta daisy (*Leucanthemum* × *superbum*). (d) Expression of *TcEbFS*‐*GUS* promoter fusions in transgenic chrysanthemum. d1, a whole leaf; d2, a longitudinal section of a leaf vascular bundle (vb); d3 and d4, cross‐sections of a shoot. (e) Pyrethrum sections showing the specific location of terpene products and callose by purple staining with, respectively, NADI and aniline blue reagents. e1, a NADI‐stained cross‐section of flower peduncle; e2, a NADI‐stained cross‐section of upper flower peduncle; e3, a NADI‐stained cross‐section of lower flower peduncle; e4, brightfield image with white arrows indicating stained oil accumulations inside inner cortex cells; e5, fluorescence image of the same section as e4 with red arrows indicating callose of some phloem sieve plates (Se). Lignified walls show autofluorescence. e6, a longitudinal section of double‐stained material (fluorescence image) showing the vascular bundle (vb) with stained callose bands of sieve plates and alongside in elongated parenchyma cells of inner cortex trains of stained EβF oil. Co, cortex; phs, phloem sclerenchyma; ph, phloem; Se, sieve elements; xy, xylem; xys, xylem sclerenchyma.

**Figure 4:**
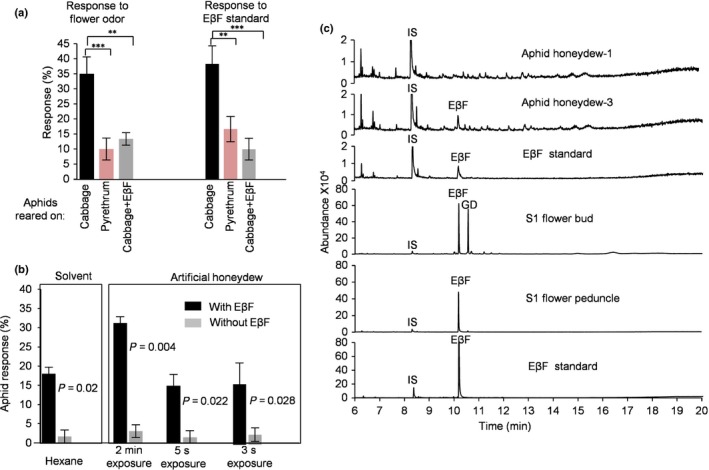
Flower‐produced and synthetic (E)‐β‐farnesene (EβF) cause alarm responses in *Myzus persicae* aphids at two levels. (a) Alarm responses to S2 pyrethrum flower odor or EβF standard (nearly 100 ng) after prerearing under different conditions. Data are represented as mean ± SEM (*n* = 6–8). Arcsin‐transformed data were subjected to ANOVA followed by Duncan's multiple range test (**, *P* < 0.01; ***, *P* < 0.001). (b) Alarm response at different exposure times to artificial honeydew (25% sucrose in water) and hexane solutions with or without 10 ng μl^−1^ EβF. Data and statistics are the same as in (a). (c) Representative total ion chromatograms of hexane extracts of pooled aphid honeydew droplets and S1 flower buds and peduncles relative to an EβF standard. Aphid honeydew‐1 is a negative sample (consisting of 50 droplets in 50 μl hexane). Aphid honeydew‐3 is a positive sample (consisting of 50 droplets in 50 μl hexane) with EβF estimated at 0.12 ng μl^−1^ (Table 2). EβF standard was 0.1 ng μl^−1^. Internal standard (IS) was carvone.

EβF emission from young flowers was dramatically promoted by mechanical damage of the peduncle, but on intact flowers nearly all EβF (27‐fold more on average) emission was derived from the flower and not by the supporting peduncle (Figs 2c, S3c). To understand production and storage of EβF, we cloned two allelic full‐length cDNAs of the *EbFS* gene from pyrethrum flower buds. The *TcEbFS1* and *TcEbFS2* open reading frames encoded two proteins of 575 and 577 amino acids respectively. They were 98% identical, with only six amino acids different and shared 93% homology with EbFS from *Artemisia annua* (Fig. S4a,b) (Yu *et al*., 2013). Fusion constructs of the *EbFS* genes in frame with GFP yielded fluorescence in the cytosol of transfected *Arabidopsis* protoplasts (Fig. S5). The genomic clone of the *EbFS* gene was 2321 bp and contained seven exons and six introns (Fig. S6). Recombinant *Tc*EbFS1 and *Tc*EbFS2 enzymes catalyzed the conversion of FPP to the single product (*E*)‐β‐farnesene (Fig. 3a). qRT‐PCR amplifying both alleles revealed that the expression of *TcEbFS* in S3 flowers was highest in the peduncle and receptacle organs (Fig. 3b), which are the parts normally most vulnerable to aphid infestation in related species (Fig. 3c). *TcEbFS* transcripts were also detected in S3 disk flowers and young leaves, but only traces of transcripts were recorded in ray flowers and older leaves (Fig. 3b). Expression peaked in S1 buds and rapidly decreased during flower development, consistent with a beetle preference for young flowers as observed in both the laboratory and the field (Figs 1b, 2d, 3b). EβF accumulated in the same organs where *TcEbFS* was most highly expressed. The peduncle stood out by uniquely accumulating EβF at the highest level with virtually no pyrethrins or lactones, whereas the flower head accumulated not only EβF but also increasingly high levels of potentially repellent pyrethrins, lactones and germacrene D (Figs 4c, S7). EβF content of the flower head decreased with flower development, but was consistently high in the flower peduncle (Fig. S7).

In summary, we observed that *EbFS* gene expression and EβF content were highest in peduncle and receptacle organs just below the young flower petals, a feeding location favored most by aphids in related plant species such as *Leucanthemum* and *Chrysanthemum* (Fig. 3c).

### EβF accumulates in inner cortex cells surrounding the vascular bundles

To reveal the tissue‐specific production and potential storage sites of EβF within a flower peduncle or a leaf, the nucleotide sequence of a 2.2 kb promoter region was cloned, fused with the *GUS* reporter gene and transformed to *Chrysanthemum morifolium* (Fig. S8). GUS staining showed that *EbFS* promoter activity was dominant in the inner cortex tissue surrounding the vascular bundles of the stem and visible only in the veins of leaves (Fig. 3d). Longitudinal sections confirmed gene expression to be located in the inner cortex cells around the vascular bundle (Fig. 3d2).

To investigate the effect of inner cortex expression on EβF accumulation and tissue localization, we quantified the EβF content in S1 flower bud and peduncle and microscopically assessed cross‐ and longitudinal sections of tissues stained for terpenes with the terpene‐specific reagent NADI (Caissard *et al*., 2004) and for callose on sieve plates with the callose‐specific aniline blue (Öner‐Sieben *et al*., 2015). EβF stored in S1 flowers amounted to 48–190 ng mg^−1^ fresh flower bud and 133–502 ng mg^−1^ S1 flower peduncle (Table 2; Fig. 4c). Largely in correspondence with the GUS staining data, NADI‐stained cross‐sections subsequently revealed abundantly stained cells filled with terpenes (presumably EβF) around the vascular bundles near the stained sieve elements (Fig. 3d,e). In longitudinal sections of the flower peduncle, NADI‐stained oils filled longitudinally connected cells surrounding the vascular system (Fig. 3e6).

**Table 2:** (E)‐β‐farnesene (EβF) content of aphid honeydew and pyrethrum flower parts.

Flower samples analyzed	EβF content^a^ (in winter)	EβF content (in spring)
S1 flower bud	48 ± 5 ng mg^−1^	190 ± 15 ng mg^−1^
S1 flower peduncle	133 ± 20 ng mg^−1^	502 ± 58 ng mg^−1^
Honeydew samples analyzed	Average EβF concentration in droplets (assumed droplet volume 100 nl)	Total EβF content per sample
Aphid honeydew‐3 (50 droplets)	1.23 ng μl^−1^	6.15 ng
Aphid honeydew‐4 (40 droplets)	0.26 ng μl^−1^	1.05 ng
Aphid honeydew‐8 (65 droplets)	0.32 ng μl^−1^	2.06 ng

a Plants used for honeydew collection.

In conclusion, EβF was stored, nearly void of other terpenes, inside cortex cells at high concentrations (~0.01–0.05% FW) (Table 2; Fig. S7). In cross‐sections only a fraction of cells visibly carried the oil, but those were usually completely filled and mainly distributed in the indented region between two vascular bundles where aphids were observed to preferentially insert their stylets. The spontaneous EβF emission from the flower head and the high levels in the flower peduncle raised the question of whether aphids could be alarmed and repelled both by directly emitted EβF from flowers before settlement, and by indirectly ingested EβF that is released via the honeydew (Tjallingii & Hogen, 1993).

### Flower volatiles and aphid honeydew cause aphid alarm responses due to EβF

To investigate why aphids were absent in pyrethrum fields, we studied the effects of flower‐emitted EβF on host‐plant acceptance. We first inoculated the pyrethrum flower head with individual *M. persicae* aphids reared on cabbage or pyrethrum and observed their behavior. Compared to aphids pre‐adapted to pyrethrum, we found that aphids reared on cabbage frequently walked up and down around the flower head during 5‐min recording periods (Fig. S9). To examine the role of EβF in the background of the other flower odors, we then performed dispersal assays on cabbage leaf disks with pyrethrum‐reared and cabbage‐reared aphids, plus aphids reared on cabbage in an EβF atmosphere. Aphids reared on cabbage were similarly alarmed by S2 pyrethrum flower volatiles and synthetic EβF, while aphids reared on pyrethrum flowers were alarmed significantly less by the headspace of S2 flowers and synthetic EβF (Figs 4a, S10). Furthermore, aphids reared on cabbage in the presence of EβF were also significantly less alarmed by both synthetic EβF and S2 pyrethrum flower volatiles. This indicates that the alarm response to pyrethrum was caused by EβF and that previous exposure to pyrethrum habituated the aphids to EβF (Fig. 4a) (de Vos *et al*., 2010).

During the preflowering stages, EβF emission was still relatively low although the peduncles had already accumulated high amounts of pure EβF (Figs 2c, S7). We suspected that the localization in the inner cortex cells surrounding the phloem sieve elements results in encounters and ingestion of EβF by sap‐sucking herbivores during the initial probing phases. To test this hypothesis, we introduced aphids (*M. persicae*) on flower peduncles (S1) and continuously collected fresh aphid honeydew droplets released during feeding. EβF was detected in three of the 19 pooled samples, but the content of the three EβF‐containing samples varied by more than a factor of 6 even for subsequent collections from the same aphids feeding on the same flower. This suggested that aphids only occasionally excrete honeydew droplets containing EβF (< 1/40) and at variable concentrations depending on the amount of cortex cell content ingested (Fig. 4c; Table 2). No EβF was detected in any of 11 similarly pooled samples of honeydew produced on the host plant *N. benthamiana* used for maintaining the aphid population, which supports the assumption that the pyrethrum plant is the origin of EβF in honeydew. To assess the effect of such EβF concentrations in honeydew on aphids, we prepared artificial honeydew containing 10 ng μl^−1^ EβF and performed an aphid behavior assay. We found that 33% of aphids exposed to this artificial honeydew (*n* = 60) showed an alarm response within 2 min when a droplet with 2 ng EβF was deposited on their dorsum, which was significantly higher than the response to droplets without EβF (*P* < 0.01) (Fig. 4b). However, normally the honeydew droplets after production are rapidly kicked away. To mimic this condition, we hovered the droplet with 2 ng EβF on a pipette very close above the dorsum of settled aphids but retracted after just 3 or 5 s. Fifteen per cent of the aphids subsequently dispersed within 2 min independent of the duration of exposure, which was significantly more than aphid response to droplets without EβF (*P* < 0.05) and comparable with the alarm effect caused by 2 ng pure EβF in hexane (18% responding aphids) (Fig. 4b).

## Discussion

Our data show that pyrethrum plants exhibit a specific defensive mimicry against aphids based on the aphid alarm pheromone EβF that effectively protects their most valuable organs: the flowers. The production of EβF is tissue‐ and developmental stage‐specific. Constitutive emission from the flower head repels aphids and attracts their ladybird predators. Moreover, upon ingestion of EβF, the aphids could release it in their honeydew excretions and function as an alarm. Defense of flowers in a tritrophic context has rarely been studied, although flowers are the most important plant organs for Darwinian fitness. Cowpea flowers emit caterpillar‐induced volatiles that attract a parasitoid enemy of the caterpillars (Dannon *et al*., 2010). However, the defensive mimicry strategy of pyrethrum flowers has a double‐edged effect as it both repels the herbivorous aphid and attracts its ladybird predator simultaneously.

For an aphid alarm pheromone produced by plants to be effective it needs to mimic the purity and dynamics of the natural aphid pheromone and direct predators to the potential location of prey (Joachim *et al*., 2013; Vosteen *et al*., 2016). Here, we took a comprehensive field‐observation‐based tritrophic approach including a plant, aphid and predator. In most natural, tritrophic studies documented to date, aphid alarm pheromone‐producing plants required induction or release by colonizing aphids (Harmel *et al*., 2007; Verheggen *et al*., 2013). This may help predators to locate prey, but fails to directly repel aphids already at the host‐selection stage, and also risks virus transmission and adaptation to the (slowly) induced cue (Hatano *et al*., 2008). Pyrethrum flowers appear to implement an ingenious EβF‐based aphid‐effective defense system. First, the abundant visitation of beetle predators in flowering pyrethrum fields is associated with chemotypes with higher EβF emission as an attractive component and specific developmental stages with comparatively more dominant levels of EβF emission and the lowest contents of pyrethrins. Beetle visitation and top‐down searching behavior on the young pyrethrum flowers is expected to serve a bodyguard function if EβF‐habituated aphids initiate a colony. Second, and probably more important is that the absence of aphids in pyrethrum fields could be correlated with repellence of aphids by EβF emitted by flowers.

This aphid‐specific defense system is emulated by specific accumulation of EβF around the vascular system of young flower peduncle and receptacle organs. At the tissue and cellular level a subsection of longitudinally stretched inner cortex cells surrounding the phloem was found to be completely filled with highly pure EβF oil. The cells were reminiscent of laticifers that are normally filled with polymeric latex terpenoids (Pickard, 2007). A question is how the release of EβF from the flowerhead is achieved. In pyrethrum S1 flowers, we observed the initiation and filling of secretory cavities around the vascular bundles. These may have enabled upward transport of EβF to the flower head for emission (unpublished data). Such specialized cavity structures may prevent cytotoxicity of high concentrations of EβF (Lange, 2015; Turner *et al*., 2019). Active transport out of the cortex cells or desintegration of those cells may lead to a release of EβF into the lumen of these cavities.

This specific expression mode may be crucial to the effectiveness of the trait in the field, and completely different from transgenic plants with constitutive CaMV 35S‐regulated EβF emissions or natural herbivory‐induced emissions (Zhuang *et al*., 2012; Bruce *et al*., 2015). The engineered examples were successful in some laboratory settings (Beale *et al*., 2006), but failed to attract higher numbers of predators or parasitoids, or to reduce aphid populations in other laboratories (Kunert *et al*., 2010; de Vos *et al*., 2010) or field trials (Bruce *et al*., 2015). This may point to the importance of EβF emission at the appropriate developmental stage and tissue. EβF was mainly emitted by pyrethrum flower heads and was less masked by germacrene D at the early flowering stages that are most sensitive to aphids. It proved sufficient to recruit beetles as bodyguards, but by a narrow developmental time margin, which coincided with a usual vulnerability to aphids.

Ladybird beetles were dominantly found on S2 flowers in the pyrethrum field. Beetle preference for the younger flower stages was confirmed in the laboratory. The specificity may be explained by the fact that at that stage EβF was least mixed with other plant volatiles that are known to interfere with alarm pheromone recognition. Alternatively, it is also possible that the increasing levels of pyrethrins in later stages can be detected by the beetles and deter them. Additionally, one may speculate that the evolutionary success of this trait may have depended on some reward to retain attracted, but ‘cheated’ beetles for as long as possible. For example, S2 disk flowers already produce nectar accessible to the beetles (Prasifka *et al*., 2018). To explain beetle preference for S2 flowers, we further assume that concentration gradients must have played a role, as the emission of EβF from the S2 flower head was about 27 times stronger than from the peduncle (Fig. S3c).

To explain the general lack of aphids in fields of flowering pyrethrum in contrast to related species, where peduncles of young flowers are the most preferred feeding location (Fig. 3c), we imagine that in the field the exposure of aphids to pure bouts of EβF is dynamically controlled by diffusion, air movements, flower density and flowering stage. These jointly create the stochastic variation in concentrations that could effectively provide false alarm to aphids. In our alarm‐response assays we found a fraction of ~35% peach aphids responding to pyrethrum flower volatiles. This is in line with studies reporting 20–40% of peach aphids responding to 50–500 ng synthetic EβF (Bayendi Loudit *et al*., 2018). It is common across species that only a fraction of the aphids respond to alarm pheromones, which probably balances the advantages of sustaining the colony with the risks of starting a new one (de Vos *et al*., 2010). Future studies with this plant species should also take into account the possibility that the chemotype diversity for EβF and pyrethrins as observed in field populations might be key in promoting species richness and the abundance of herbivore and/or predator communities (Ninkovic *et al*., 2011). Certainly, the observed practice of Chinese farmers in Yunnan province to use this plant for intercropping in orchards and vegetable gardens suggests that the plant can fulfil such a function.

Next to the spontaneous emission from the flower head, the storage of EβF oil in cortex cells close to the sieve elements suggested an additional layer of more direct biological interaction with those aphids that failed to respond to flower‐emitted EβF. Those aphids will engage into test‐probing inner cortex cells in search of sieve elements and encounter at some frequency cells filled with EβF (Tjallingii & Hogen, 1993). This may lead to some EβF ingestion and subsequent release in the honeydew, but only during that early searching phase. Indeed, upon examining pools of collected honeydew droplets, we observed an expected low frequency of ingestion and secretion of pyrethrum EβF. We assume that as we found EβF in 3/19 analyzed pools, and as it does not accumulate in sieve elements, that it was derived from just one or two droplets that contained cortex cell content. A droplet of 100 nl could then contain all EβF found in the pooled samples and 1.0–6.2 ng EβF per droplet (Table 2). Interestingly, this is in the published range of amounts released by stressed aphids attacked by ladybird beetles or lacewing larvae reported as 0.25–2.5 ng for peak emissions per 2 min and 9.5–15 ng for total emissions per 2 h (Schwartzberg *et al*., 2008; Joachim *et al*., 2013). Honeydew droplets are only briefly resident on an aphid before they are kicked away with their legs, but in terms of anatomy the honeydew‐releasing anus is very close to the large placoid (LP) sensilla on the sixth antennal segment that detect EβF when they are folded backward when feeding (Zhang *et al*., 2017). We show that EβF at concentrations found in honeydew droplets can within seconds set off a false alarm in aphids in addition to the effects of the flower headspace. Thus, the pattern of EβF accumulation around the phloem and occurrence of functional amounts of EβF in the honeydew suggests a functional role also at this more intimate level.

Studying defense mechanisms of flowers is crucial to understand how plants safeguard offspring production (Zangerl & Berenbaum, 2009). The double‐edged mechanism revealed here not only provides insight into the multifaceted defense of pyrethrum flowers, but also shows how effective such defense can be in the field. Gene editing studies in which components of the system have been knocked out will even more directly highlight the interconnections and investigating flower defenses in general will be important for the development of effective crop protection, especially for seed crops.

## Author contributions

MAJ and CW initiated and supervised the research project; JL, HH, CW and MAJ designed the experiments; JJL, MAJ, HH, JM, LY, GS and MW performed research; JL, MAJ, HH, GS, NCAdR, MD and RM analyzed data; JL, MD and MAJ wrote the manuscript; JL and MAJ contributed equally to this work.

## Supporting information

Please note: Wiley Blackwell are not responsible for the content or functionality of any Supporting Information supplied by the authors. Any queries (other than missing material) should be directed to the *New Phytologist* Central Office.

**Fig. S1** Headspace composition of flowers with different levels of ladybird beetle presence
**Fig. S2** Volatiles emitted from intact pyrethrum plants at different developmental stages
**Fig. S3** Volatile compounds in the headspace of pyrethrum flowers and leaves after mechanical wounding and herbivore infestation
**Fig. S4** Alignment and phylogenetic analysis of the deduced amino acid sequences of plant‐derived (*E*)‐β‐farnesene synthases
**Fig. S5** Subcellular localization of *TcEbFS* gene expression using transient expression of a GFP fusion protein
**Fig. S6** Exon/intron organization of *EbFS* genes from *T. cinerariifolium* and three other plant species
**Fig. S7** Analysis of secondary metabolites in pyrethrum flower head and peduncle at different developmental stages
**Fig. S8** Alignment of the promoter sequences of *(E)‐*β*‐farnesene synthase* genes of *T. cinerariifolium* and *A. annua*
**Fig. S9** Aphid movement on early‐stage pyrethrum flowers in the field
**Fig. S10** Alarm effect of *M. persicae* aphids reared on pyrethrum and cabbage
**Methods S1** Survey of entomofauna
**Methods S2** Headspace collection and volatile analysis of field samples by thermo‐desorption GC‐MS
**Methods S3** Headspace collection and analysis of intact pyrethrum plants and on‐plant analysis of a single leaf upon mechanical damage by GC‐MS
**Methods S4** Olfactory responses of ladybird beetles to different odors
**Methods S5** Isolation, characterization and functional expression of *E*β*F synthase* genes and promoter sequence from *T. cinerariifolium*
**Methods S6** Plant secondary metabolite extraction and analysis
**Methods S7** Aphid behavior assay in response to early‐stage pyrethrum flowers
**Methods S8** Aphid honeydew collection and volatile analysis
**Table S1** DNA oligonucleotide primers used in this research
