# Supplementary material for: Defense of pyrethrum flowers: repelling herbivores and recruiting carnivores by producing aphid alarm pheromone
Source: New Phytol. 2019 May 31;223(3):1607–20. doi: 10.1111/nph.15869 (PMC6772172; doi:10.1111/nph.15869)
Supplement: Supplementary file 1 — Fig. S1 Headspace composition of flowers with different levels of ladybird beetle presence Fig. S2 Volatiles emitted from intact pyrethrum plants at different developmental stages Fig. S3 Volatile compounds in the headspace of pyrethrum flowers and leaves after mechanical wounding and herbivore infestation Fig. S4 Alignment and phylogenetic analysis of the deduced amino acid sequences of plant‐derived (E)‐β‐farnesene synthases Fig. S5 Subcellular localization of TcEbFS gene expression using transient expression of a GFP fusion protein Fig. S6 Exon/intron organization of EbFS genes from T. cinerariifolium and three other plant species Fig. S7 Analysis of secondary metabolites in pyrethrum flower head and peduncle at different developmental stages Fig. S8 Alignment of the promoter sequences of (E)‐β‐farnesene synthase genes of T. cinerariifolium and A. annua Fig. S9 Aphid movement on early‐stage pyrethrum flowers in the field Fig. S10 Alarm effect of M. persicae aphids reared on pyrethrum and cabbage Methods S1 Survey of entomofauna Methods S2 Headspace collection and volatile analysis of field samples by thermo‐desorption GC‐MS Methods S3 Headspace collection and analysis of intact pyrethrum plants and on‐plant analysis of a single leaf upon mechanical damage by GC‐MS Methods S4 Olfactory responses of ladybird beetles to different odors Methods S5 Isolation, characterization and functional expression of EβF synthase genes and promoter sequence from T. cinerariifolium Methods S6 Plant secondary metabolite extraction and analysis Methods S7 Aphid behavior assay in response to early‐stage pyrethrum flowers Methods S8 Aphid honeydew collection and volatile analysis Table S1 DNA oligonucleotide primers used in this research [file NPH-223-1607-s001.pdf]

***New Phytologist* Supporting Information**

Article title: **Defense of pyrethrum flowers: repelling herbivore and recruiting carnivore by producing aphid alarm pheromone**

Authors: Jinjin Li, Hao Hu, Jing Mao, Lu Yu, Geert Stoopen, Manqun Wang, Roland Mumm, Norbert C.A. de Ruijter, Marcel Dicke, Maarten A. Jongsma, Caiyun Wang

Article acceptance date: 11 April 2019

The following Supporting Information is available for this article:

Fig. S1 Headspace composition of flowers with different levels of ladybird beetle presence

Fig. S2 Volatiles emitted from intact pyrethrum plants at different developmental stages

Fig. S3 Volatile compounds in the headspace of pyrethrum flowers and leaves after mechanical wounding and herbivore infestation

Fig. S4 Alignment and phylogenetic analysis of the deduced amino acid sequences of plant-derived (*E*)-β-farnesene synthases

Fig. S5 Subcellular localization of *TcEbFS* gene expression using transient expression of a GFP fusion protein

Fig. S6 Exon/intron organization of *EbFS* genes from *T. cinerariifolium* and three other plant species

Fig. S7 Analysis of secondary metabolites in pyrethrum flower head and peduncle at different developmental stages

Fig. S8 Alignment of the promoter sequences of *(E)-β-farnesene synthase* genes of *T. cinerariifolium* and *A. annua*

Fig. S9 Aphid movement on early stage pyrethrum flowers in the field

Fig. S10 Alarm effect of *M. persicae* aphids reared on pyrethrum and cabbage

Table S1 DNA oligonucleotide primers used in this research

Methods S1 Survey of entomofauna

Methods S2 Headspace collection and volatile analysis of field samples by thermo-desorption GC-MS

Methods S3 Headspace collection and analysis of intact pyrethrum plants and on-plant analysis of a single leaf upon mechanical damage by GC-MS

Methods S4 Olfactory responses of ladybird beetles to different odors

Methods S5 Isolation, characterization and functional expression of *EβF synthase* genes and promoter sequence from *T. cinerariifolium*

Methods S6 Plant secondary metabolites extraction and analysis

Methods S7 Aphid behavior assay in response to early stage pyrethrum flowers

Methods S8 Aphid honeydew collection and volatile analysis

Fig. S1 Headspace composition of flowers with different levels of ladybird beetle presence

Comparison of representative total ion chromatograms of the headspace of flowers with and without beetles using detached flowers from the fields. Indicated compounds are those statistically having higher emission rates in flowers with or without beetles respectively (Table 1). (*Z*)-3-hexen-1-ol (HO), (*Z*)-3-hexenyl acetate (HA) and (*E*)-β-farnesene (EβF) were the dominant volatiles in the headspace of the detached flowers. A '+' indicates that this compound has a higher abundance in flowers with/without beetles and the percentage relative to the ion abundance value of flowers without beetles (See Table 1).

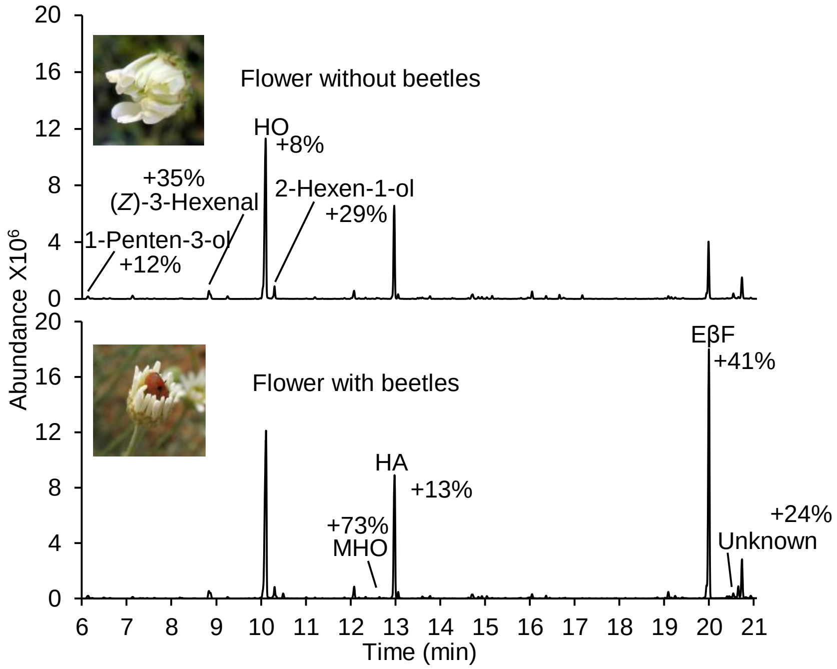

Fig. S2 Volatiles emitted from intact pyrethrum plants at different developmental stages

(a) Representative total ion chromatograms of the headspace of whole pyrethrum plants at different developmental stages (pre-flowering, S0-2, S2-4, S4-6, see Fig. 2*A*). Peaks were identified as Camphene (1), (*Z*)-3-Hexen-1-ol acetate (2, HA), Isolongifolene (3), β-Longipinene (4), (*E*)-β-farnesene (5, EβF), Germacrene D (6, GD), respectively. Peak 7 is the internal standard (7) methyl laurate.

(b) Semi-quantitative comparison of the six most abundant volatiles emitted during plant development. Data are means ± SE of three biological replicates and expressed as peak area (TIC).

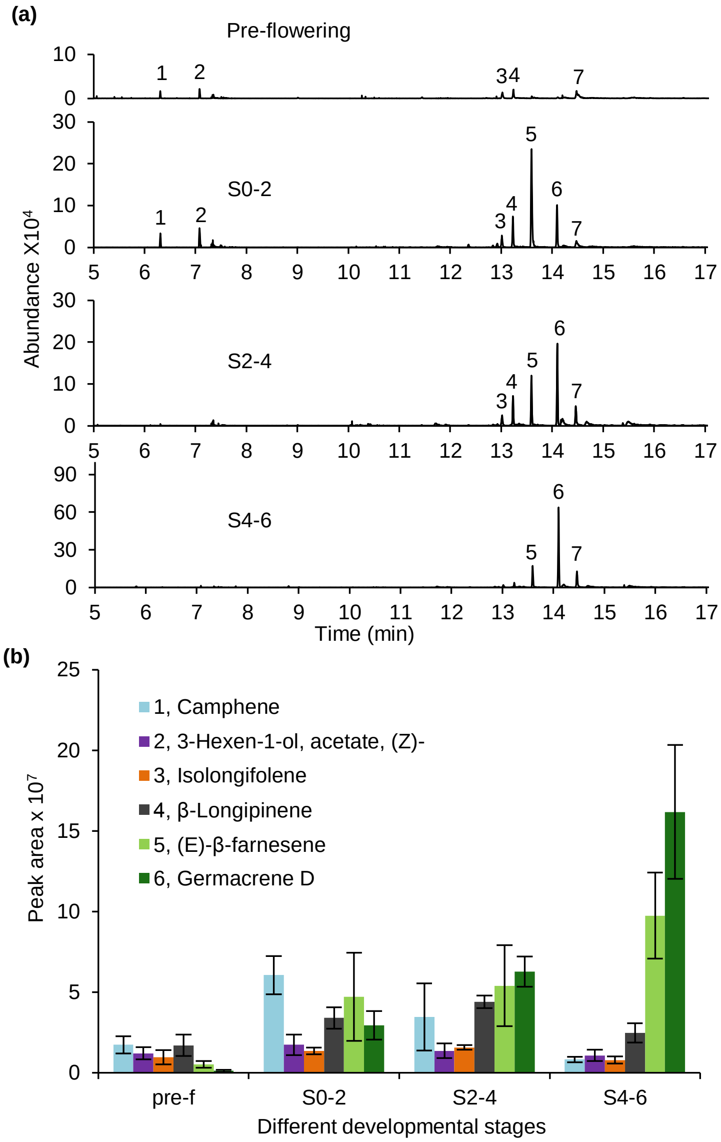

Fig. S3 Volatile compounds in the headspace of pyrethrum flowers and leaves after mechanical wounding and herbivore infestation

(a) Representative total ion chromatograms of headspace of pyrethrum leaves, either intact or after mechanical wounding. (*Z*)-3-hexen-1-ol (HO), (*Z*)-3-Hexen-1-ol acetate (HA) and (*E*)-β-farnesene (EβF).

(b) Temporal pattern of emission of headspace volatiles emitted by wounded leaves while attached to non-flowering pyrethrum plants in mounted flasks during subsequent 30 min time intervals: 5-35 min, 45-75 min, 85-115 min and 125-155 min after mechanical damage. The scale of the much higher EβF peak is shown on the right axis. Data were expressed as peak areas (TIC). Error bars indicate means ± SE of three biological replicates.

(c) Changes in HO, HA and EβF emission of five pooled S2 flower peduncles (buds removed the day before), S2 flowers, S2 flowers infested with 10 aphids (fl + aphids) or 20 thrips (fl + thrips) each for 2 hours. The values are expressed as peak areas of the major masses 67, 82, and 69 for HO, HA and EβF, respectively. Data are means ± SE (n = 3). Pre indicates pretreatment headspace collection of 2 hrs and 2 hrs indicates a similar headspace collection of two hours immediately after insect inoculation. Significance is relative to the flower control after a log data transformation for the peduncle and relative to the pre-inoculation data for the insect treatments (t-test: * = $p < 0.05$, ** = $p < 0.01$).

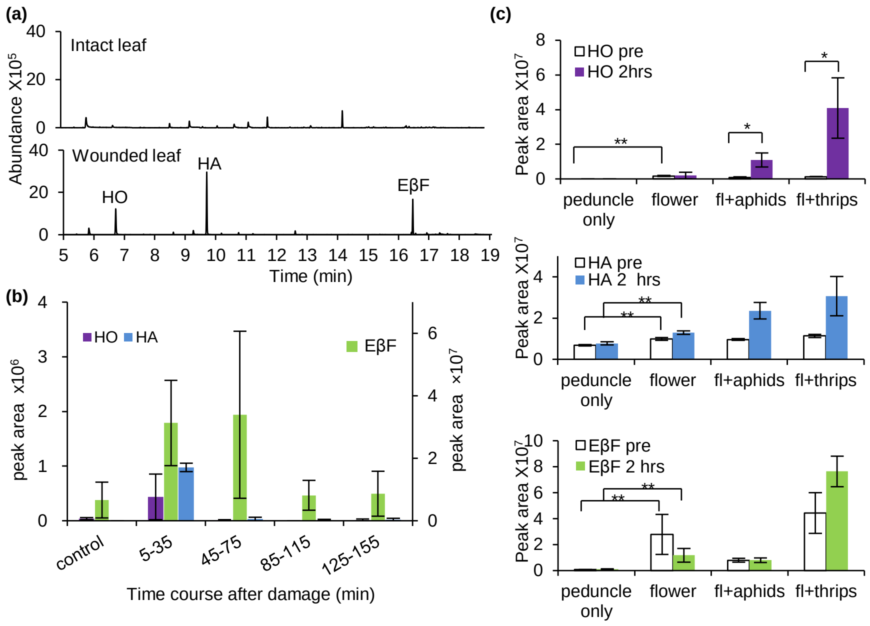

Fig. S4 Alignment and phylogenetic analysis of the deduced amino acid sequences of plant-derived (*E*)-β-farnesene synthases

(a) Alignment of the deduced amino acid sequences of (*E*)-β-farnesene synthases. Alignments were performed with Clustal Omega (http://www.ebi.ac.uk/Tools/msa/clustalo/) and were shaded with BoxShade version 3.21 software (http://www.ch.embnet.org/software/BOX_form.html). (*E*)-β-farnesene synthase amino acid sequences were from *Pseudotsuga menziesii* (PmEBF, AAX07265); *Pinus sylvestris* (PsEBF, ADH29869); *Mentha arvensis* (MrEBF, ADC92564); *Zea mays* (ZmEBF, Q2NM15); *Mentha asiatica* (MsEBF, AEA49038); *Mentha* × *piperita* (MpEBF, CAH10289); *Citrus junos* (CjEBF, AAK54279); *Triticum urartu* (TuEBF, EMS64422); *Artemisia annua* (AaEBF, AAX39387); and *T. cinerariifolium* (TcEBF1 and TcEBF2), respectively. The highly conserved RXR (at positions 291 to 294 in EBF1 and 293 to 296 in EBF2) and DDXXD regions (at positions 328 to 333 in EBF1 and 330 to 335 in EBF2) were boxed.

(b) Phylogenetic analysis of plant-derived (*E*)-β-farnesene synthases and gene structure. Amino acid alignment using ClustalW was used to reconstruct the joint enrooted tree through MEGA5 by bootstrap neighbor-joining method. (*E*)-β-farnesene synthase accession numbers were the same as in **Fig. S4a**.

(a)

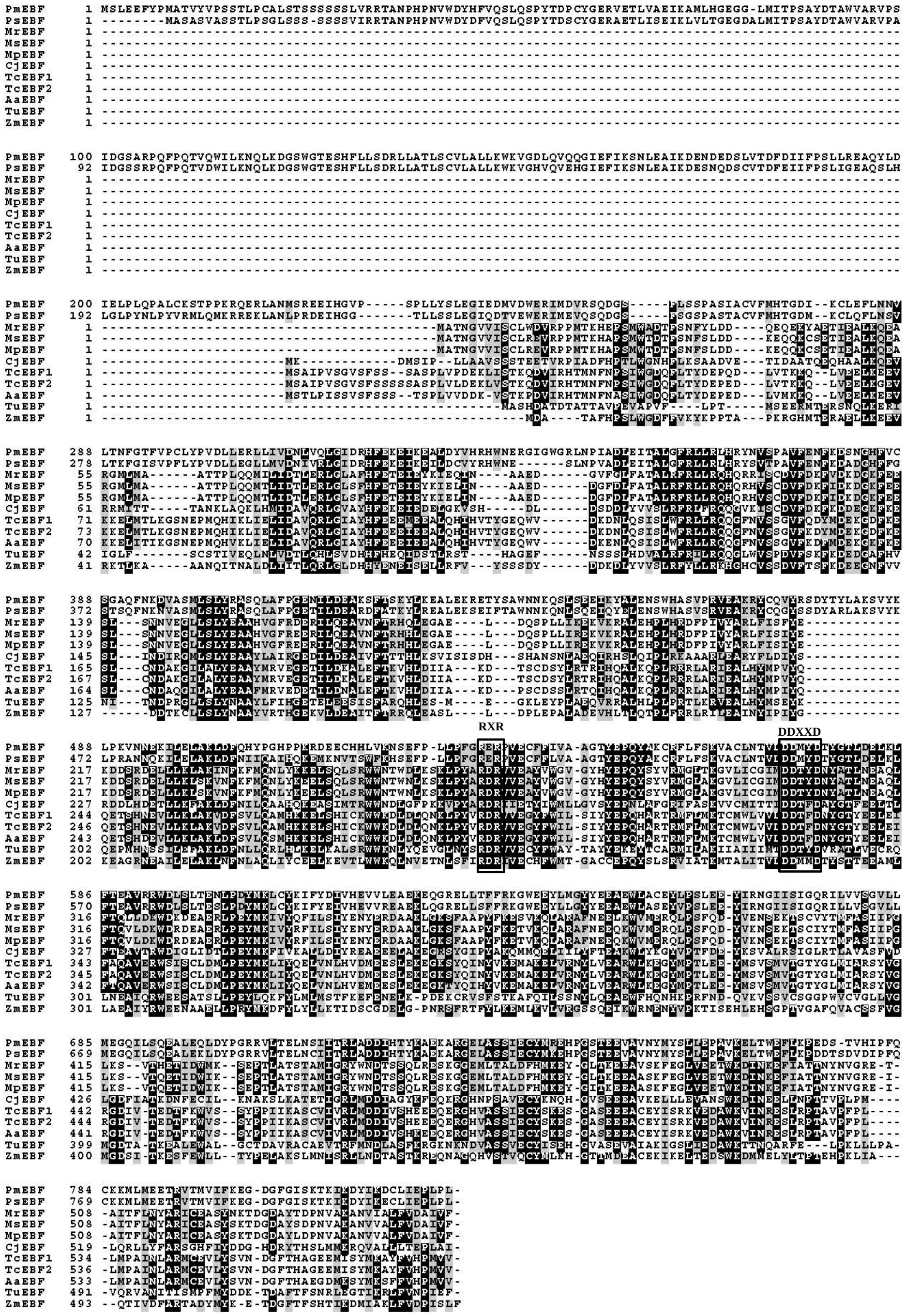

(b)

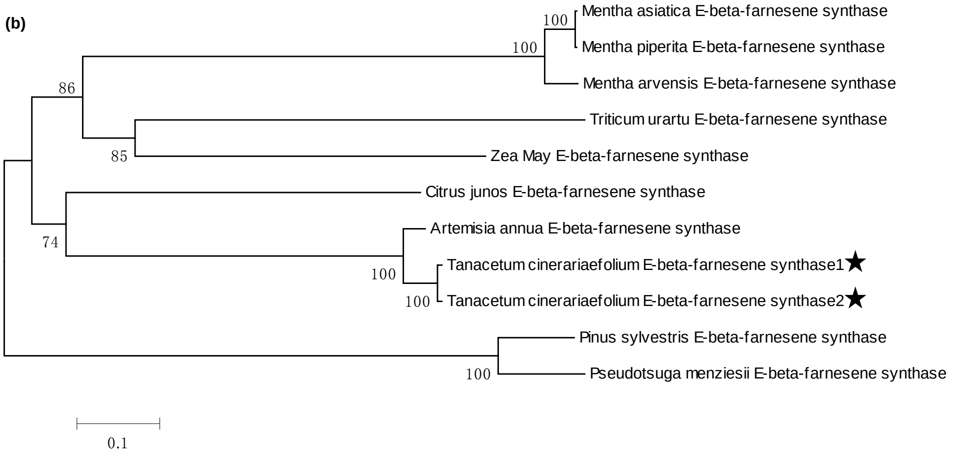

Mentha asiatica E-beta-farnesene synthase
Mentha piperita E-beta-farnesene synthase
Mentha arvensis E-beta-farnesene synthase
Triticum urartu E-beta-farnesene synthase
Zea May E-beta-farnesene synthase
Citrus junos E-beta-farnesene synthase
Artemisia annua E-beta-farnesene synthase
Tanacetum cinerariaefolium E-beta-farnesene synthase1★
Tanacetum cinerariaefolium E-beta-farnesene synthase2★
Pinus sylvestris E-beta-farnesene synthase
Pseudotsuga menziesii E-beta-farnesene synthase
100
100
86
85
74
100
100
100
0.1

Fig. S5 Subcellular localization of *TcEbFS* gene expression using transient expression of a GFP fusion protein

GFP fluorescence, bright field and merged images of Arabidopsis (*Arabidopsis thaliana*) protoplasts**.** *Tc*EbFS1-GFP and *Tc*EbFS2-GFP represent EbFS1 and EbFS2 fused to GFP localization in the cytosol; wild type, non-transformed control. GFP control, GFP expressed without fusion protein targeting with localization in the cytosol.

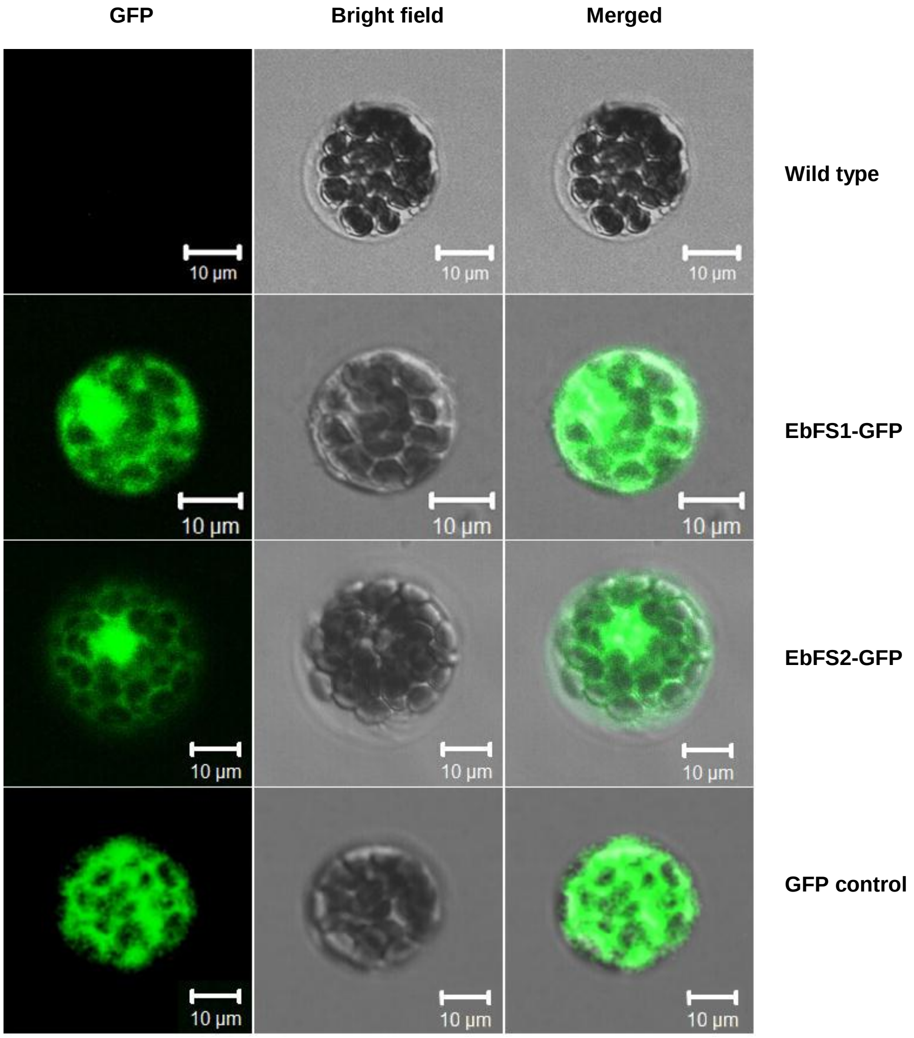

Fig. S6 Exon/intron organization of *EbFS* genes from *T. cinerariifolium* and three other plant species

Exons are marked in black boxes, introns as lines. The lengths of exon/intron fragments are shown in base pairs (bp).

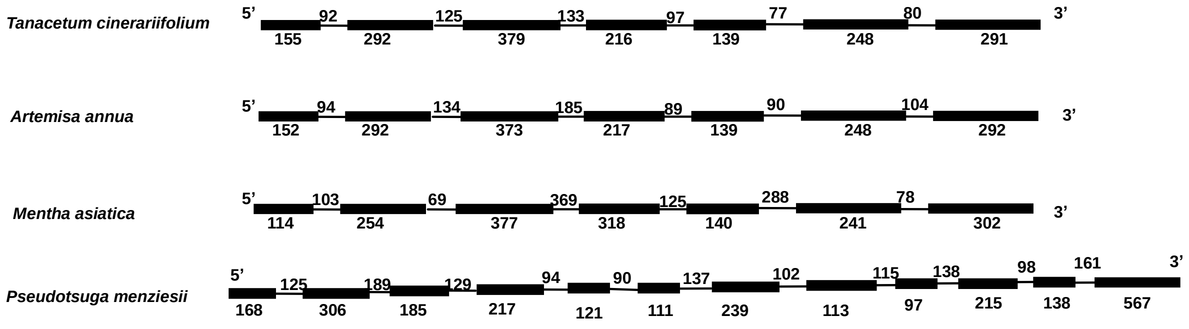

Fig. S7 Analysis of secondary metabolites in pyrethrum flower head and peduncle at different developmental stages

GC-MS analysis of the relative concentrations (relative to its highest concentration per compound) of EβF, sesquiterpene lactone and pyrethrins (based on the pyrethrin I GCMS peak) in different organs at different developmental stages. Maximum content (100 %) of EβF was 134 μg/g FW (in peduncle 3) and 7.6 mg/g FW (in flower 5) for total pyrethrins. Numbering of peduncles and flowers followed the developmental stages. Error bars indicate ± SEM of three biological replicates.

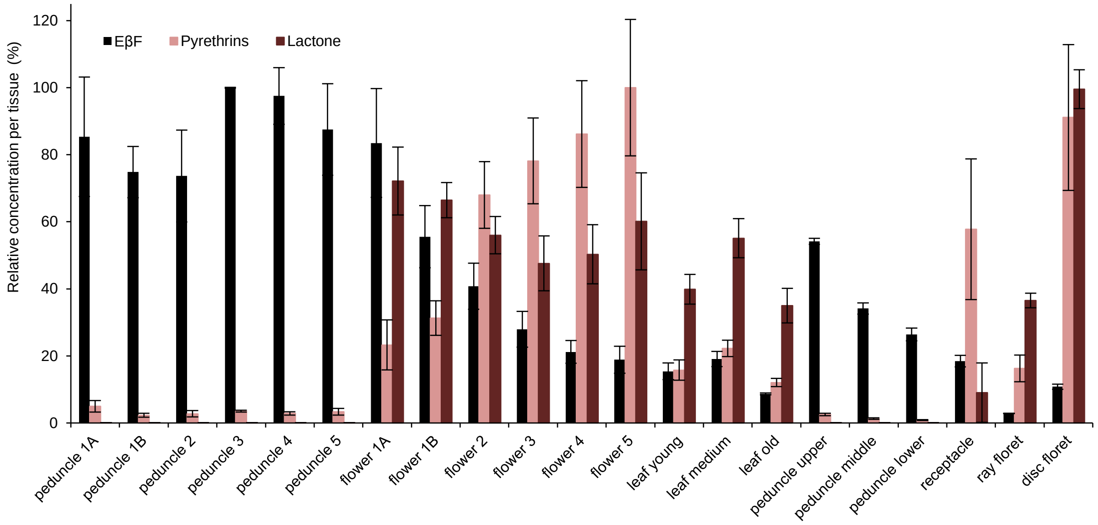

Fig. S8 Alignment of the promoter sequences of *(E)-β-farnesene synthase* genes of *T. cinerariifolium* and *A. annua*

Alignments were performed with ClustalW (http://www.genome.jp/tools/clustalw/) and were shaded with BoxShade version 3.21 software. The 1.5kb upstream sequence of (*E*)-β-farnesene synthase gene (Aa-P*EbFS*, gi|523967140|) was from *A. annua*. The sequences with bright yellow background are part of the coding sequence.

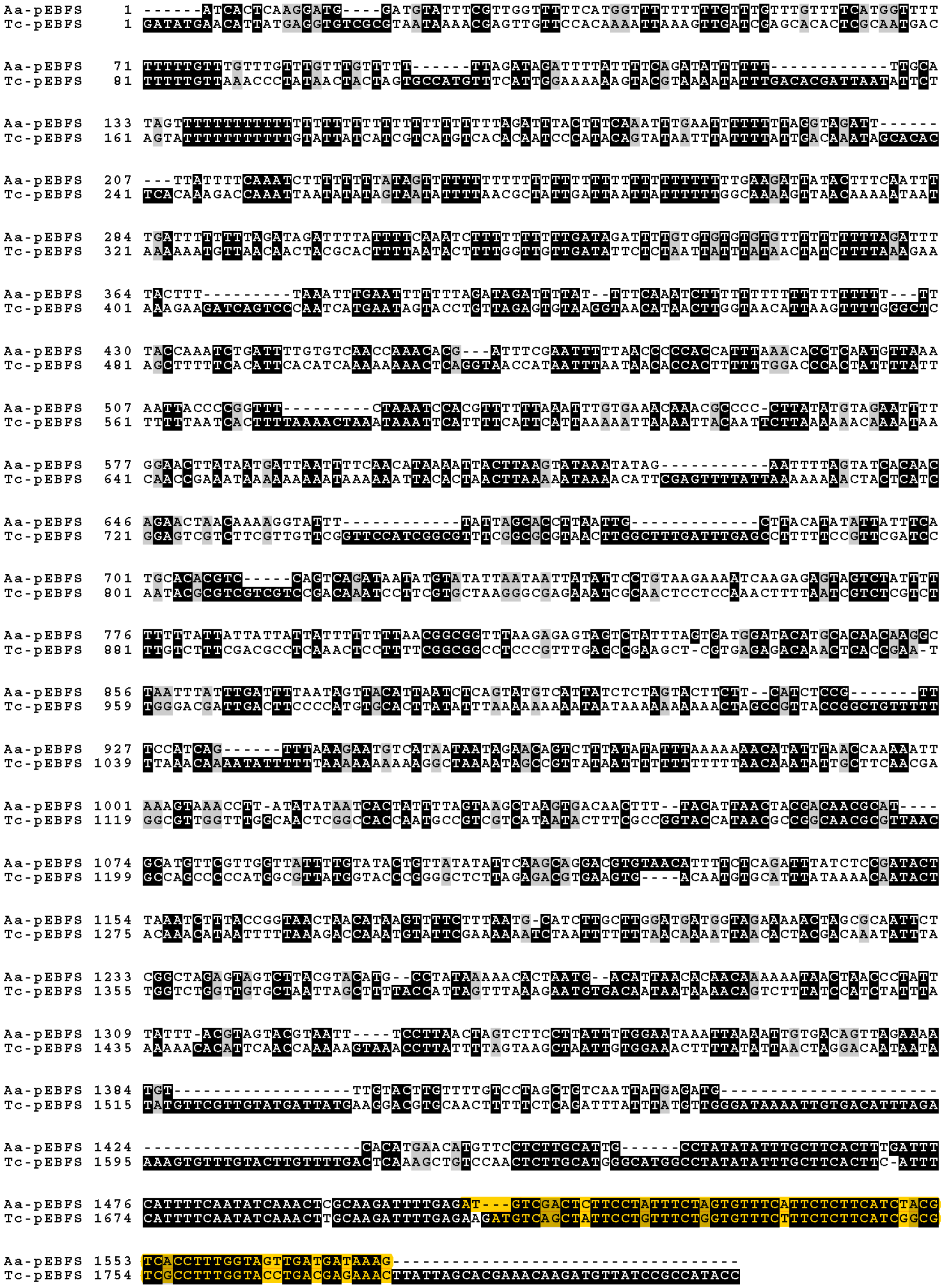

Fig. S9 Aphid movement on early stage pyrethrum flowers in the field

Aphids reared on cabbage and pyrethrum plants were individually used to observe their first response behavior by recording their position on a pyrethrum flower every five seconds. Early stage flowers (S1, S1/2, S2) from the same genotype were used to do the assay. In total sixty aphids (twenty aphids for each stage flower) were tested. Y axis represents the movements of aphids on the flower at different recording times (X axis). 0 represents the joint part of the flower head and flower peduncle; 1 represents the upper receptacle part; 2 represents the upper ray floret part; - 1 represents the lower flower peduncle.

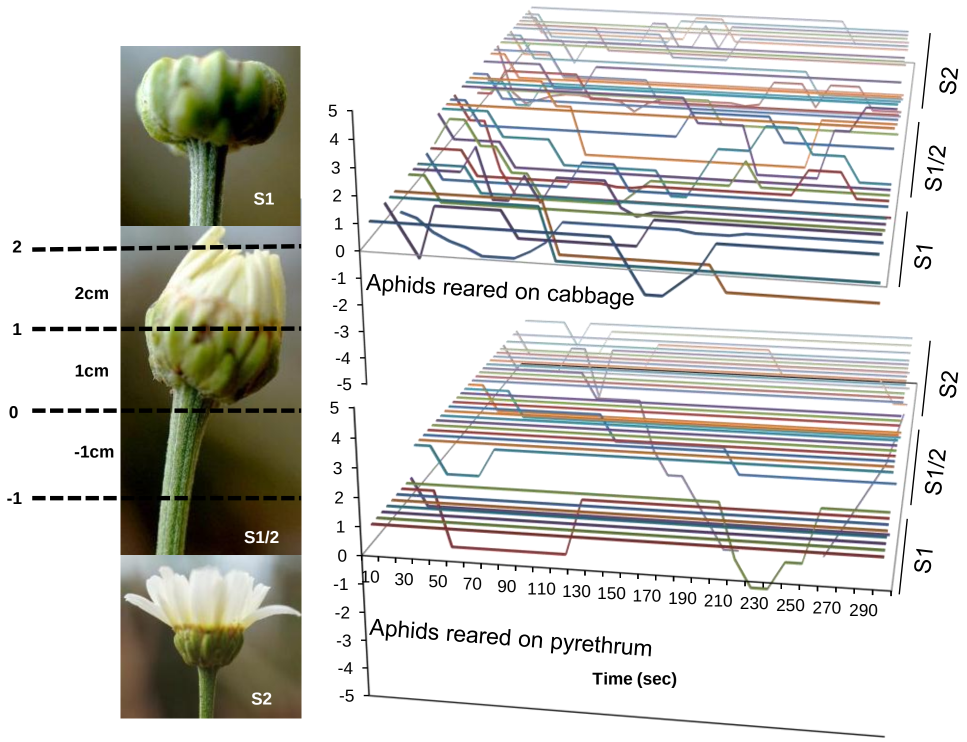

Fig. S10 Alarm effect of *M. persicae* aphids reared on pyrethrum and cabbage

Aphids reared on cabbage plants respond significantly more to the pyrethrum flower odor and 1 μg synthetic EβF compound than to clean air or hexane control, respectively. Pyrethrum-reared aphids and cabbage-reared aphids habituated to EβF were not significantly repelled by the flower odor or synthetic EβF when compared with their own control (the grey bar). A response was scored if the aphid walked away within 2 minutes from a settled state on a cabbage leaf. Values are the percentage mean ± SEM (n = 6). Arcsin-transformed data were subjected ANOVA and followed by Duncan's multiple range test. Significance is black bar relative to grey bar (* = $p < 0.05$, ** = $p < 0.01$)

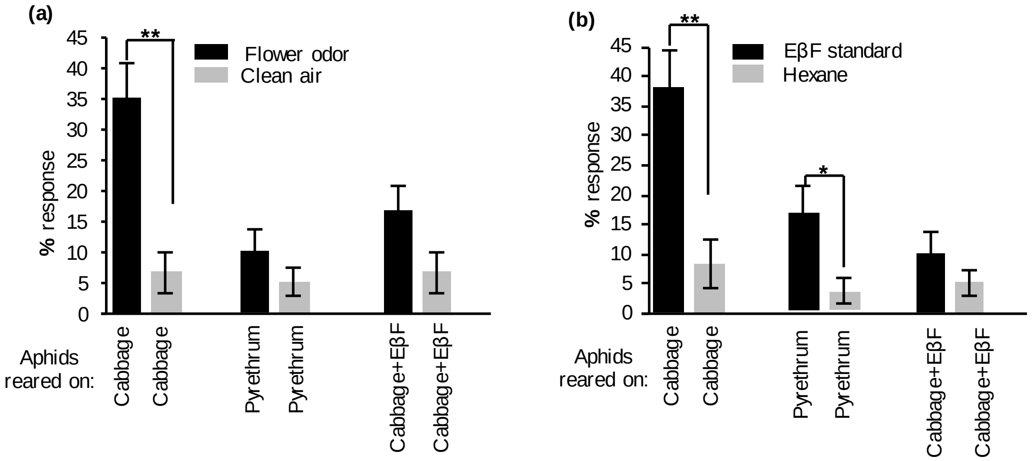

Table S1 DNA oligonucleotide primers used in this research

Function of the primers	Name of the primers	Primer sequences（5’→3’）
Amplification of the fragment	EβF-F	ACCCAAATACACCAAGCGTT
	EβF-R	CCTTTTCAAGTGATTCCTCCAT
5'-RACE	EβF5-GSP1	GTTCGAGCATGTTGAGGCTCGTAATAGATGGA
	EβF5-GSP2	CAACGCCTCTATCCTTGCCAGCCTTCT
	EβF5-GSP3	CATCTTCCACCCTCATAAATGCCGCTTC
3'-RACE	EβF3-GSP1	AGAAGGCTGGCAAGGATAGAGGCGTT
	EβF3-GSP2	AGCGATGCACAAAAAGGAGCTTAGCC
Adaptor primer of RACE	UPM-Long	CTAATACGACTCACTATAGGGCAAGCAGTGGTATCAACGCAGAGT
Full-length of ORF and Genome DNA of *EβFS*	EβF-ORF-F	TTGAGAAGATGTCAGCTATTCCTG
	EBF-ORF-R	TTAGACAACCATAGGGTGAACGA
PET-EβFS	EβF-PET-F	TCTAAGCTTGCGAATTCTTCAGCTATTCCTGTTTCTG
	EβF-PET-R	GCGGCCGCCAGAATTCGACAACCATAGGGTGAACGAAG
Pcambia1302-EβFS	EβF-1302-F	CATGCCATGGTATCAGCTATTCCTGTTTCTG
	EβF-1302-R	GGACTAGTGACAACCATAGGGTGAACGAAG
anti-*EβFS*1	1st FNIP-PCR	GCTGACGTAGGAGTCGAAACCAAAGTG
anti-*EβFS*2	2st FNIP-PCR	CGACCCACTGCTCACCATATGTTAC
anti-*EβFS*3	3st FNIP-PCR	GGTATGGCGGATAACATCTTGTTTCGTGC
PBI121-PEβF-GUS	EβF-PBI121-F	CAAGATTTTGAGAAGGGATCCATGTCAGCTATTCCTGTTTCTGGTG
	EβF-PBI121-R	CGATCGGGGAAATTCGAGCTCTTAGACAACCATAGGGTGAACGAAG
	PE-F	GATTATGAAGGACGTGCAAC
	GUS-R	GCTTTCTTGTAACGCGCTTGAGT
Real-time PCR	RT-EβF-F	TTTCTTTCTCTTCATCGGCGTC
	RT-EβF-R	CGTAACCAAATCTTGTGGCTCA
	RT-GAPDH-F	AGACGAGTTTCACAAAGTTG
	RT-GAPDH-R	AGGAATCTGAAGGCAAGC

Methods S1 Survey of entomofauna

Fields of 0.2 - 0.5 ha near villages Mulong 24°27'10.34"N-10 3°32'21.01"E and Alufa 24°34'20.57"N-103°44'53.58"E in Luxi county, Yunnan province) were monitored for the presence and behavior of different insect species during the flowering periods of spring 2009, 2010 and 2015. In 2010, a large-scale survey was carried out in China by visually monitoring insects in three fields, in three rows of 100 plants per field (in total 900 plants). The data on the two neighboring fields near Mulong with similarly high ladybird beetle frequencies covering 600 plants were used in this report. The other field had a 20-fold lower density of beetles which was too low to include for statistical analysis. Monitoring was done each week for 3 consecutive weeks from a stage when flowers were predominantly in stage 2 to finally predominantly stages 4-5.

Methods S2 Headspace collection and volatile analysis of field samples by thermo-desorption GC-MS

A metal tube (140 × 4 mm) filled with Tenax adsorbent (200 mg Tenax; 20/35 mesh; Markes Ltd) was connected to the inlet to purify incoming air. A second Tenax cartridge was connected to the outlet to trap volatiles. Headspace volatiles from different samples were collected at an airflow of approximately 100 ml/min using an aquarium pump to push the air through two serially connected cuvettes with two Tenax cartridges in between. Serial connection was done to ensure identical collected volumes between sample pairs because the flow from the pump was known to be not completely regular under those field conditions. The first cuvette contained 10 freshly harvested S2 flowers with 10 cm peduncles derived from 10 independent plants on which a beetle had been observed, and the second cuvette contained 10 S2 flowers from 10 independent plants from the same row without beetles visiting.

For analysis of volatiles released by the peduncle, whole flower, with and without thrips or aphid herbivory, on day 1 (15:00 hrs) 60 S2 flowers of mixed genotypes were harvested from the field. Flower peduncles were all cut to the same length (20 cm) and randomly placed in 12 sets (4 treatments) of five in 100 ml cuvettes containing 80 ml water. From three sets the flower buds were removed leaving the peduncle only. At 10:40 on day 2, from all 12 sets a pretreatment headspace was collected for two hours after first overlaying the water with 20 ml paraffin oil to prevent any volatiles from the cut peduncle to be released via the water. Each cuvette was placed

inside a 2.5 l glass container with tubes filled with Tenax connected at the inlets and outlets as described above for thermodesorption GC-MS except that in this laboratory set-up all collections were in parallel at 100 ml per minute. Next, 20 onion thrips (*Thrips tabaci*) and 10 green peach aphids (*M. persicae*) per flower were inoculated on two times three sets of flowers and immediately at 13:40 a second 2-hour headspace collection was started for the four treatments (peduncles, flowers, flowers plus aphids and flowers plus thrips) by placing the flowers and peduncles back into the containers. Quantitation was done by calculating the peak area of the main specific mass for each compound ((*Z*)-3-hexen-1-ol (HO): 67; (*Z*)-3-hexenyl acetate (HA): 82; (*E*)-β-farnesene (EβF): 69). Significance analysis was done by t-test.

Methods S3 Headspace collection and analysis of intact pyrethrum plants and on-plant analysis of a single leaf upon mechanical damage by GC-MS

The collection vessels were sealed 10-liter glass containers with two connector plugs on the top. Headspace was collected for 24 h (12 h light and 12 h dark). Volatiles were eluted from the liner in 1 ml hexane (HPLC grade) and then methyl laurate (8.7 ng/μl, Sigma-Aldrich, Co., LLC., USA) was added as an internal standard and analyzed by GC-MS on a Thermo Trace GC (Thermo Fisher Scientific, USA) coupled to a DSQ mass-selective detector (Thermo Fisher Scientific, USA). Separation was performed on a HP-5MS column (30 m × 0.25 mm i.d. × 0.25 μm film thickness, Agilent Technologies, USA) with helium as the carrier gas. The detector voltage was 1700 V, and ion spectra were obtained with 70 eV with a scan range from 40 to 700 atomic mass units with a data acquisition rate of 20 Hz. One μl of eluate was injected in splitless mode into the GC at an injection port temperature of 280 ℃. The GC oven ramp was 45 ℃ for 1 min, 10 ℃ min^{-1} to 280 ℃, and held there for 3.5 min. The transfer line temperature was 220 ℃.

A single leaf was enclosed in a 50 ml glass extraction bottle with its petiole sticking through the hole of a soft cap (HM-0020A, http://www.nbhmyq.com/cn/index.php) preventing tissue damage and gas exchange. Bottles were replaced with clean ones at the end of each interval. Volatiles were collected by inserting a purged clean SPME fiber into the bottle through the lids for 30 min at room temperature. For the mechanical damage treatment, each time half of a fully expanded leaf of a 3-month-old genotype ‘39’ pyrethrum was cut off to create a wounded surface. The fiber was injected manually and desorbed in the injection port of the GCMS system as

described（Zeng *et al.*, 2016）. Volatiles were collected from the damaged leaf during five subsequent periods until EβF release had returned to its original level.

References

Zeng XL, Liu C, Zheng RR, Cai X, Luo J, Zou JJ, Wang CY. 2016. Emission and accumulation of monoterpene and the key terpene synthase (TPS) associated with monoterpene biosynthesis in *Osmanthus fragrans* Lour. *Frontiers in Plant Science* **6**:1-16.

Methods S4 Olfactory responses of ladybird beetles to different odors

At least four potted, greenhouse-grown pyrethrum plants of each development stage were individually placed in a 10 l glass flask linked by Teflon (PTFE) tubes to the two arms of Y-tube olfactometer, which had an 18 cm trunk and 9 cm arms and a 60° angle between the two arms and the pots were wrapped in aluminum foil. The Y-tube was placed with the two arms facing upwards at an angle of 45° to allow for negative geotaxis common in ladybirds (Oliveira & Pareja 2014). Compressed air was passed through active charcoal and bubbled through water at a flow rate of 500 ml/min before entering the exposure chamber. The Y-tube olfactometer was positioned inside a box accessible from above and closed by a lid (30 × 30 × 30 cm) to avoid visual disturbance. The only light source was a cold light lamp (Schott KL 1500 LCD, 2950K) placed above the center of the Y tube.

Each C. septempunctata, starved for 6h at room temperature, was introduced into the base of the Y-tube and observed for 5 min. If the individual entered 2 cm into one of the arms and remained in that arm for at least 30 s, it was considered to have made a choice, and the bioassay was terminated. If an individual did not make a choice within 10 min, it was considered a non-responder. After every five insects tested, the sources of the arms of the Y-tube were exchanged and after every ten insects tested, the Y-tube was exchanged for a new one. The Y-tubes were each time cleaned with pure ethanol, rinsed with distilled water, and dried in an oven.

EβF (Bedoukian Research Inc. Danbury-CT, USA, product nr P3500-90; 96 % pure), (*Z*)-3-hexen-1-ol (HO), (*Z*)-3-hexenyl acetate (HA) and 6-methyl-5-hepten-2-one (MHO) were purchased from Sigma Aldrich and made in paraffin oil (Sigma Aldrich, prod. no. 18512) and quantified by collecting the headspace from closed cuvettes. On this basis it was determined that the headspace of field collected S2 flowers with beetles could be mimicked by solutions of pure

or blended 1 % (v/v) EβF, 0.0005 % (v/v) MHO, 0.07 % (v/v) HA and 0.06 % (v/v) HO in paraffin oil.

References

Oliveira MS, Pareja M. 2014. Attraction of a ladybird to sweet pepper damaged by two aphid species simultaneously or sequentially. *Arthropod-Plant Interactions* **8**:547-555.

Methods S5 Isolation, characterization and functional expression of *EβF synthase* genes and promoter sequence from *T. cinerariifolium*

Total RNA and genomic DNA from pyrethrum S1 flower buds were isolated using Trizol (CoWin Biotech Co., Ltd, Beijing, China) according to the manufacturer's instructions and CTAB method. Then, 1 μg total RNA was reverse transcribed with an oligo (dT) primer using the TransScript II One-Step gDNA Removal and cDNA Synthesis SuperMix (TransGene Biotech CO., Ltd, Beijing, China). Based on the sequence of the *A. annua* (*E*)-*β-farnesene synthase* gene (Picaud *et al.*, 2005), specific primers (listed in the Table S1) were designed to amplify the core fragment of *EbFS* from pyrethrum. PCR amplification was done according to the procedure of 2x Es Taq MasterMix (CoWin Biotech Co., Ltd, Beijing, China). For elongation of the 5' and 3' cDNA ends of the *EbFS* gene, the cDNA was synthesized with primers (listed in the Table S1) containing an adaptor sequence at the 5'-end. Both 5' and 3' rapid amplification of cDNA ends (RACE) PCR were performed with gene-specific primers (listed in the Table S1, designed with the core fragment information) with the SMART-RACE PCR kit (Clontech, Mountain View, CA, USA) according to the manufacturer's instructions. The full open reading frame and genomic DNA of the *EbFS* gene were amplified by PCR using cDNA (*TcEbFS1* and *TcEbFS2* sequences deposited in GenBank under accession number: MF678594 and MF678595) and gDNA (sequences deposited in GenBank under accession number: MF682058). A new high-efficiency strategy for rapid chromosome walking PCR (Wang *et al.*, 2011) was used to amplify the upstream sequence of the *EbFS* gene by the adaptor and specific primers (anti-*EbFS*1, anti-*EbFS*2 and anti-*EbFS*3, see in the Table S1), three rounds of PCR were performed. All the PCR products were cloned into pEASY-T1/T5 vector (TransGene Biotech CO., Ltd, Beijing, China) and transformed into competent *E. coli* T1 according to the manufacturer's instructions. Positive colonies were checked by individual bacterial colony PCR and selected for sequencing by Tsingke (Wuhan, China). DNA sequence data were assembled and analyzed using DNAMAN

software (LynnonBiosoft, USA) and putative amino acid sequences were analyzed in GenBank databases using the NCBI BLAST program. Schematic structures of *EbFS* were drawn in a gene structure display server (GSDS, http://gsds.cbi.pku.edu.cn/). The theoretical isoelectric points (pI) and molecular weights (MW) of the proteins were computed using the Compute pI/MW Tool (http://web.expasy.org/compute_pi/). Alignment of the deduced protein sequences was performed using DNAMAN and CLUSTAL_X version 1.83. A joint unrooted phylogenetic tree was constructed by MEGA5 using the neighbor-joining method.

The complete open reading frame of the full-length cDNA of *EbFS* was subcloned in-frame and upstream of the (His)6-tag of the pET6xHN-C expression vector by introducing an *Eco*RI site on the forward primer and excision of the stop codon in the reverse primer with the same *Eco*RI site. Plasmids were transformed into BL21 (DE3) *E. coli* cells and verified by sequencing. For bacterial expression, *E. coli* DE3 harboring pET6xHN-C (bearing a full-length insert) and empty pET6xHN-C plasmids were grown overnight at 37 ℃ in a starter culture (10 ml Luria-Bertani medium with 50 mg/l Amp). A portion of the starter culture (1 ml) was then diluted in 50 ml LB medium with 50 mg/l Amp to OD_{600} = 0.6. The cells were induced by adding 1 mmol/l IPTG for 18 h at 18 ℃. Cells were then collected by centrifugation at 3000 g for 15 min. The harvesting and purification of the recombinant proteins were done according to the HisTALON™ Gravity Column Purification Kit User Manual (Clontech, www.clontech.com).

References

Picaud S, Brodelius M, Brodelius PE. 2005. Expression, purification and characterization of recombinant (*E*)-β-farnesene synthase from *Artemisia annua*. *Phytochemistry* **66**:961-967.

Wang Z, Ye S, Li J, Zheng B, Bao M, Ning G. 2011. Fusion primer and nested integrated PCR (*FPNI-PCR*): a new high-efficiency strategy for rapid chromosome walking or flanking sequence cloning. *BMC Biotechnology* **11**:1-12.

Methods S6 Plant secondary metabolites extraction and analysis

Frozen tissues were ground to powder and transferred to 500 μl hexane in a glass tube (containing 1.67 ng/μl carvone as an internal standard). The hexane containing tissue powder was vortexed for 30 s and followed by 5 min of sonication. The extracts were centrifuged for 10 min at 3000 *g* and the supernatant was dried by passing it through a column filled with Na_2SO_4.

Secondary metabolites stored in the different organs at various plant developmental stages were extracted. Sampling was done as follows. For extraction of flower parts from one genotype, stage 2 flowers and 3 × 3 leaves (young, medium, old) were harvested. Pools of 5 flowers derived from 5 different plants were dissected into 4 cm subsequent peduncle sections (upper, middle, lower, starting right under the receptacle), receptacles, ray florets and disc florets. For flower development, 9 genotypes were used to harvest 9 flowers of all flowering stages (S1-5). Three flowers were used per replication. Young S1 flowers were subdivided into two developmental stages of a small bud (1A) and a bud (1B) immediately prior to opening. Each detached flower was dissected into a peduncle part (12 cm below bud) and a flower part (the bud). All materials were folded into aluminum sheeting and immediately frozen in liquid nitrogen and weighed. A Retsch bullet grinder was used to grind the samples. 100 mg of material was extracted in 2 ml of dichloromethane (DCM) and vortexed for 15 sec, subsequently centrifuged at 3000 rpm for 15 sec and the supernatant was passed through a column filled with sodium sulphate to remove water. Flow through was collected in glass 2 ml vials and used for GC-MS analysis as described before (Ramirez *et al.,* 2012). The identification and relative quantitation of pyrethrins and lactone were performed by comparing with commercial pyrethrin oil (2 mg/ml, Honghe Senju Biological, China) and using a selective mass ion (specific mass 123 is used for pyrethrins and specific mass 43 for lactones). EβF was identified and quantified by comparing with a dilution series of an EβF standard.

References

Ramirez AM, Stoopen G, Menzel TR, Gols R, Bouwmeester HJ, Dicke M, Jongsma MA. 2012. Bidirectional secretions from glandular trichomes of pyrethrum enable immunization of seedlings. *The Plant Cell* **24**:4252-4265.

Methods S7 Aphid behavior assay in response to early stage pyrethrum flowers

A pyrethrum field with nearly ten hundreds flowering pyrethrum plants (mostly S0-S3 stages flowers) planted in a company yard (Yuxi, Yunnan province, China) was used to do this experiment. Three early-stage (S1, S1/2, S2) pyrethrum flowers were selected for observation of aphid response behavior. Aphids at fourth instar or young adult reared on cabbage plants or pyrethrum flowers were individually used to observe their first-response behavior by continuously recording their position on a pyrethrum flower every five seconds. The total

recording lasted for five minutes. For each experiment, aphids reared on cabbage plants or pyrethrum flowers were individually tested. Each aphid was used one time on one flower. In total, sixty cabbage-reared and sixty pyrethrum-reared aphids (twenty aphids for each flower stage) were tested by releasing them individually on the top of a flower head (S1) or the ray florets (S1/2, S2). We defined the joint part of the flower head and flower peduncle as 0; 1 represents the upper receptacle part; 2 represents the upper ray floret part; - 1 represents the lower flower peduncle and record aphid position every five seconds. The data are presented in a plot showing aphid position on the flower as a function of time of recording. The Y axis shows the aphid position and X axis shows time. Aphids were excluded from the result data if they directly walked to the lower part of the flower peduncle.

Pyrethrum flower volatiles were collected by inserting five S2 flowers from one genotype into the neck of a 500 ml headspace jars without cutting the flower peduncles and sealing it by fitting moistened cotton wool around the peduncle. The jars were sealed 500 ml glass containers with two connector plugs on the top and bottom. EβF or hexane solvent control was introduced by adding 1 µl on a filter paper disc and sealing it into the jars for 2 h. A 50 ml plastic syringe was connected to the jar via a metal tube containing Tenax TA at the inlet to clean the entering air. By pressing the syringe, an amount of 50 ml clean air was blown into the jar and the headspace of the test substances was blown out through the upper Teflon outlet tubing. To check for unforeseen asymmetries, a standard solution of hexane alone and clean air were included as two control treatments. For each test, a fresh aphid settled on a cabbage leaf in a Petri dish with moistened absorbent cotton was used. 10 aphids were repeated for each 500 ml jar. In total 60 aphids reared on pyrethrum and 60 aphids reared on cabbage plants were tested for each odor.

Methods S8 Aphid honeydew collection and volatile analysis

Fresh honeydew droplets were collected by placing the plants horizontally so that the flower peduncles with aphids were hovering just above a large glass Petri dish (Ø = 15 cm). Landing droplets were picked up immediately using a micro-capillary with rubber balloon for suction and pressure and deposited into 50 µl hexane (containing 1.67 ng/µl carvone). A minimum of 40 droplets were collected. For the control, aphids were inoculated on fresh *N. benthamiana* plants and honeydew was collected in the same way. The volume of the droplets was calculated by assuming a half sphere shape and measuring the average droplet diameter.

One microliter of pooled aphid honeydew extract was injected in splitless mode on an Agilent 7890A GC coupled to an Agilent 5973 mass selective detector. The GC was equipped with an HP-5MS (Agilent Technologies, USA) capillary column (30 m × 0.25 mm i.d. × 0.25 μm). The GC oven temperature program was initiated at 45 °C, held for 2.25 min then raised with 40 ℃ min^{-1} to 300 ℃ with 5 min hold. Other operating conditions were as follows: He carrier gas with a constant flow rate of 1 ml/min; injector temperature, 260 ℃. Mass spectra were obtained by electron impact at 70 eV.
